# Functional phylosymbiosis in birds: phylogeny and antimicrobial properties of bacterial symbionts in Bucerotiformes

**DOI:** 10.1186/s42523-026-00608-y

**Published:** 2026-08-07

**Authors:** María Dolores Barón, Manuel Martín-Vivaldi, Ester Martínez-Renau, Raphaël Arlettaz, Derek Engelbrecht, Charlotte Enkvist, Elbert Lukas Jansen van Rensburg, Lu Zhang, Antonio Martín-Platero, Manuel Martínez-Bueno, Ángela Martínez-García, Cristina Ruiz-Castellano, Michael Schaub, Mark Stanback, Tanja M. F. N. van de Ven, Elin Videvall, Wei Liang, Juan José Soler

**Affiliations:** 1 Departamento de Ecología Funcional y Evolutiva, Estación Experimental de Zonas Áridas (EEZA-CSIC), Almería, E-04120 Spain; 2 Departamento de Zoología, Facultad de Ciencias, Universidad de Granada, Granada, E-18071 Spain; 3 Unidad Asociada (CSIC): Coevolución: Cucos, Hospedadores y Bacterias Simbiontes, Universidad de Granada, Granada, E-18071 Spain; 4 Department of Ecology and Genetics, Uppsala University, Uppsala, Sweden; 5 Division of Conservation Biology, Institute of Ecology and Evolution, University of Bern, Bern, 3012 Switzerland; 6 Department of Biodiversity, University of Limpopo, Sovenga, 0727 South Africa; 7 Department of Biochemistry, Microbiology and Biotechnology, University of Limpopo, Sovenga, 0727 South Africa; 8 Ministry of Education Key Laboratory for Ecology of Tropical Islands, College of Life Sciences, Hainan Normal University, Haikou, 571158 China; 9 Departamento de Microbiología, Universidad de Granada, Granada, E-18071 Spain; 10 Swiss Ornithological Institute, Sempach, 6204 Switzerland; 11 Department of Biology, Davidson College, Davidson, North Carolina United States; 12 Mogalakwena Research Centre, Alldays, South Africa; 13 Brain Function Research Group, School of Physiology, Faculty of Health Sciences, University of the Witwatersrand, Johannesburg, South Africa

**Keywords:** 16S-rRNA gene sequencing, Antimicrobial activity, Microbiome, Phylosymbiosis, Preen gland, Symbiotic bacteria, Uropygial secretion

## Abstract

Bacterial symbionts and their animal hosts differ in the strength and type of selection pressures that promote their evolution and, as some host traits are phylogenetically conserved, their symbiotic bacterial communities could also exhibit greater similarity than expected by chance; a pattern known as phylosymbiosis. This might result from differences in bacterial assemblages across species-specific habitats, and/or from natural selection favouring host traits that allow harbouring functional species-specific bacteria (functional phylosymbiosis). We explored these patterns in eight Bucerotiformes taxa, including hoopoes, woodhoopoes, and hornbills, of which hoopoes and woodhoopoes harbour antibiotic-producing symbionts in their uropygial gland. We characterised uropygial secretion bacterial communities using high-throughput 16S-rRNA gene sequencing and analysed its association with avian phylogeny. Furthermore, we quantified antimicrobial activity of bacterial isolates, and explored its relationship with host phylogenetic distances and bacterial community composition. We found a significant correlation between avian phylogeny and bacterial community dissimilarities, supporting phylosymbiosis. Moreover, antimicrobial activity was significantly predicted by bacterial community composition and the relative abundance of particular strains in hoopoes and woodhoopoes. These findings suggest that antimicrobial properties of symbionts could be responsible for the detected phylosymbiosis and that natural selection may favour uropygial gland features allowing hosts to harbour specific antimicrobial-producing symbionts.

**Supplementary information:**

The online version contains supplementary material available at https://doi.org/10.1186/s42523-026-00608-y.

## Introduction

The evolution and diversification of life are deeply shaped by interspecific interactions and, in some cases, the relationships between species become so intimate that the evolution of one drives the evolution of the other [1, 2]. Among interactions driving animal diversity, those between animals and bacteria have emerged as one of the most important [3, 4]. Bacterial symbionts provide hosts with multiple benefits, including pathogen defence [5, 6], immune system modulation [7], and nutrient acquisition [8], suggesting that they have likely played a key role in animal evolution [9, 10]. Animal traits that might have evolved because of their interactions with beneficial bacteria are those that facilitate the acquisition and cultivation of particular symbionts [11–13]. Hosts’ behavioural, morphological, or physiological characteristics that facilitate the colonisation of beneficial bacterial communities, as well as efficient parent-offspring transmission, would be rapidly selected for in host populations [9]. Thus, host evolution is likely to parallel changes in the diversity and composition of the symbiotic bacterial communities, or in the evolution of particular bacterial strains, which might even be the result of specialisation through coevolutionary processes. This is likely the case in fungus-growing ants, where specialised morphological host structures (i.e., crypts and exocrine glands) have evolved to harbour symbiotic bacteria that protect the ants’ fungal crops from pathogens [14].

Congruent or similar clustering between the phylogeny of hosts and the composition of their symbiotic bacterial communities is termed phylosymbiosis, and may or may not reflect evolutionary patterns [15, 16]. Phylosymbiosis can arise through stochastic mechanisms, such as ecological drift, dispersal events, or hosts’ interspecific differences in environmental conditions (e.g., habitat characteristics, geographic distribution), or by deterministic mechanisms, including host-driven selection, ecological niche differentiation, co-diversification, or co-speciation [17]. To explore the possibility that a functional microbiome is the consequence of host evolution, detecting signals of phylosymbiosis should be seen as a first step [16]. It would further require understanding the functional role of symbionts that confer fitness advantages to their hosts. Most evidence for host traits allowing the evolution of beneficial microbiome (adaptive evolution) in scenarios of phylosymbiosis comes from transplant experiments under controlled laboratory conditions [16]. Some of these works compare fitness-related traits of hosts that were transplanted with conspecific or heterospecific microbiomes, and detected negative effects of heterospecific transplants [18, 19]. This suggests that species-specific microbiomes are associated with host fitness [15], but not necessarily indicate that phylosymbiosis has evolved as a consequence of the benefits derived from host’s symbionts (functional phylosymbiosis). Detecting that the benefits provided by bacterial symbionts vary across host species and correlate with host phylogenetic distances would suggest functional phylosymbiosis. In accordance, Brooks AW, *et al.* [15] demonstrated that when transplants of gut microbiota were conducted between phylogenetically distant species, the reduction in host fitness was more pronounced than for closely related species. These results suggest parallel evolution of the microbiome’s functionality and the phylogeny of their animal hosts.

Apart from estimating the negative effects of symbionts from other host species (sometimes referred to as allochthone symbionts), the functionality of the host bacterial communities could also be measured in terms of abundance and diversity of bacterial features responsible for the beneficial effects. This approach is particularly interesting in systems where bacteria provide hosts with antimicrobial compounds against a diverse range of pathogenic microbes. Risk of infection and breadth of potential pathogens typically vary interspecifically depending on species-specific ecological contexts, such as climate, social behaviour, or habitat characteristics, and select for host defences, including immune responses [20, 21]. Accordingly, host immune systems tend to reflect these species-specific ecological conditions [22]. Consequently, if host microbiomes are associated with preventing infections, the antimicrobial potential of these communities should also vary across species facing different infection risks [23].

Here we measure the antimicrobial activity of avian bacterial communities to explore functional phylosymbiosis in six species of the order Bucerotiformes and the microbiome of their uropygial secretion. Bacterial symbionts of the uropygial gland of birds contribute to host antimicrobial defence across diverse species [23], with the families Upupidae (hoopoes) and Phoeniculidae (woodhoopoes) providing the best-known examples of symbiotic bacteria with a potential defensive role against avian pathogens and parasites [24–26]. We characterise both the bacterial communities and the antimicrobial activity of the uropygial secretion of one species of Upupidae (including three subspecies), two Phoeniculidae, and three Bucerotidae species. Symbiotic bacteria are responsible for the distinctive dark colouration, strong odour, and antimicrobial activity of the uropygial secretions of hoopoes and woodhoopoes [25, 27, 28]. Thus, within a framework of functional phylosymbiosis, Bucerotiformes is an ideal group to explore associations between bacteriological and antimicrobial properties of the uropygial secretion. First, we tested (i) whether closely related species harbour more similar bacterial communities than distantly related species (i.e., phylosymbiosis). Moreover, as a first prediction of functional phylosymbiosis, we also explored (ii) whether interspecific differences in antimicrobial properties of secretion isolates correlate with host phylogenetic distances. Finally, (iii) we explored the association between bacterial beta diversity and antimicrobial properties, as well as identified the bacteria potentially responsible for the detected associations.

## Methods

### Study species and areas

We conducted the study using several breeding populations across Europe, Africa, and Asia. We sampled three subspecies of hoopoes (*Upupa epops*). The European hoopoe (*Upupa epops epops*) was studied in two locations (Spain and Switzerland), the African hoopoe (*Upupa epops africana*) in Namibia, and the Asian hoopoe (*Upupa epops longirostris*) in China. We included two species of Phoeniculidae. The green woodhoopoe (*Phoeniculus purpureus)* was sampled in Namibia and South Africa, and the common scimitarbill (*Rhinopomastus cyanomelas*), represented by one female, in Namibia. Hereafter, the term “woodhoopoes” refers collectively to both species, the green woodhoopoe and the common scimitarbill, but we specify the species name when necessary to distinguish between them. From the family Bucerotidae, we included three hornbill species of the genus *Tockus*: the southern yellow-billed hornbill (*T. leucomelas*) and the southern red-billed hornbill (*T. rufirostris*) from Namibia and South Africa, and the Monteiro’s hornbill (*T. monteiri*) from Namibia. European rollers (*Coracias garrulus*) were sampled in Spain and used as an outgroup in the study. The species taxonomic classification, country of origin, sample sizes, and the tested predictions are provided in Table 1.

**Table 1:** Host species and subspecies

Family and uropygial gland	Species/Subspecies	Country	Sample size	Sample size (antagonistic test and nests)	Analyses included
Upupidae	*Upupa epops epops*	Spain	Females (*n* = 10)	n = 17; nests = 10	1,2,3,4
			Nestlings (*n* = 10)		
		Switzerland	Females (*n* = 10)	Not tested; nests = 10	1
			Nestlings (*n* = 10)		
	*Upupa epops africana*	Namibia	Females (*n* = 2)	n = 9; nests = 3	1,2,3,4
			Nestlings (*n* = 7)		
	*Upupa epops longirostris*	China	Females (*n* = 3)	n = 20	1,2,3,4
			Nestlings (*n* = 17)		
Phoeniculiae	*Phoeniculus purpureus*	Namibia	Adults (*n* = 8)	n = 10	1,2,3,4
			Juveniles (*n* = 2)		
		South Africa	Adults (*n* = 4)	n = 4	1,2,3
			Juveniles (*n* = 2)		
	*Rhinopomastus cyanomelas*	Namibia	Female (*n* = 1)	n = 1; nests = 1	1,2,3
Bucerotidae	*Tockus leucomelas*	Namibia	Females (*n* = 11)	n = 28; nests = 14	1,2,3,4
			Nestlings (*n* = 17)		
		South Africa	Nestlings (*n* = 3)	Not tested; nests = 2	1
	*Tockus monteiri*	Namibia	Females (*n* = 9)	n = 9; nests = 9	1,2,3,4
	*Tockus rufirostris*	Namibia	Females (*n* = 6)	n = 6; nests = 6	1,2,3,4
		South Africa	Nestlings (*n* = 13)	Not tested; nests = 7	1
Coraciidae **Outgroup**	*Coracias garrulus*	Spain	Females (*n* = 7)	Not tested; nests = 12	1
			Nestlings (*n* = 17)		

The first column shows images of the uropygial gland of the three *Bucerotiformes* families, highlighting the large gland and dark secretion in hoopoes and woodhoopoes, and the pale secretion in hornbills. For each species and population, we report the number of sampled individuals of different ages, the number of those individuals with information on antimicrobial activity against indicator bacteria and the number of nests per specie (except for the green woodhoopoe that was captured using a net). In the last column, numbers refer to the following predictions: 1. Closely-related species harbour more similar bacterial communities than distantly related species. 2. Association between differences in antimicrobial activity and host phylogenetic distances. 3. Association between bacterial beta diversity and antimicrobial activity at an individual level. 4. Bacterial strains associated with estimated antimicrobial activity against the indicator bacteria.

Among these species, hoopoes and the common scimitarbill produce two types of uropygial secretions: dark and malodorous, produced by nestlings and breeding females (nesting individuals) (see Table 1); and pale and odourless, produced by males and non-breeding females, similar to those of hornbills, rollers, and other birds [28, 29]. In the case of green woodhoopoes, nesting and non-nesting individuals (both male and female) consistently produce dark and malodorous secretions. Since evidence for bacterial-mediated benefits of uropygial secretions in Upupidae and Phoeniculidae comes exclusively from individuals producing the dark secretion, in hoopoes and scimitarbills, we limited our sampling to nesting individuals. Further information on sampling locations, fieldwork, and nesting strategies is provided in ESM S1.1, while ESM S1.2 shows interspecific differences in uropygial secretion and gland morphology.

### Sampling uropygial gland secretion in the field

During nest visits and sampling, we wore new latex gloves cleaned with 70% ethanol. Before sampling, we cleaned the gland surface and surrounding area with a cotton swab moistened with 70% ethanol to prevent external bacterial contamination. For species with large papilla and a single aperture (i.e., hoopoes and woodhoopoes), we inserted a sterilised micropipette tip (1–10 µl) into the uropygial gland papilla and collected the secretion directly into a sterile microfuge tube (Fig. S2). For hornbills and rollers, we collected the secretion using a sterile microcapillary (32 mm, 10 µl) placed at the gland opening while gently pressing the gland until emptied (Fig. S2). Then we stored the microcapillary tube within a sterile microfuge tube. Field controls were periodically collected by simulating sampling procedures: pipetting air into sterile vials and exposing sterile microcapillary tubes to ambient air for 30 seconds. After collection, all samples were stored in a portable icebox at approximately 4 °C until arrival at the laboratory.

### Microbiological analysis

Within 36 h after collection, we cultured general aerobic mesophilic bacteria by spreading 100 µl of a serial dilution (from 10^−1^ to 10^−4^) of 5 µl of hoopoe and woodhoopoe uropygial secretion in PBS (Phosphate Buffered Saline) on tryptic soy agar plates (TSA; Scharlau Chemie S.A., Barcelona). For hornbills, the secretion was directly spread on the culture plates without dilution. Counts (colony-forming units, CFU) were obtained after 24 h incubation at 37 °C. Subsequently, up to five colonies from different morphologies were isolated and re-isolated to purity, aiming to include the maximum possible diversity present on each plate. Each of these isolates was used to evaluate antimicrobial activity against three indicator bacterial strains using the double-layer technique [30]: *Enterococcus faecalis* JH2-2, a commensal bacterium that can act as an opportunistic pathogen in animals, *Listeria innocua* CECT-340, a non-pathogenic species but phylogenetically closely related to pathogenic *Listeria* spp., it is usually used as an indicator bacteria in tests for antimicrobial capacity against pathogenic species as it shows a high sensitivity to a wide range of antimicrobials [31], and *Bacillus licheniformis* D13, a keratinolytic bacterium known for its feather-degrading capacity that is considered a bird pathogen [32–34]. The antimicrobial activity of each colony was measured with a digital calliper as the width of the growth-inhibition halos around the spot of isolates following previous work [23, 35]. Then, for each indicator bacteria, we averaged the inhibition values of the five isolated colonies. Moreover, to account for bacterial density, antimicrobial activity values were multiplied by the log-transformed bacterial density of the secretion. This approach implies that in samples with no bacterial growth, the antagonism value was assigned to zero. Bacterial density was estimated by standardising colony-forming unit (CFU) counts per ml (for hoopoes and woodhoopoes) or total colonies on the plate (for hornbills). Further methodological information on the antimicrobial activity estimation is available in ESM S1.3, and bacterial density for the considered species and subspecies in ESM S2.3. Sample sizes of species for which samples were cultured and tested for antimicrobial activity are provided in Table 1.

### DNA extraction and sequencing

DNA was extracted from the remaining uropygial secretion and from field controls, which had been stored in 500 µl of lysis buffer at − 18 °C after cultivation. The Modified Salting-Out Procedure described in Martín-Platero A, *et al.* [36] was used, incorporating mechanical lysis with FastPrep and the inclusion of zirconium beads. To generate 16S bacterial ribosomal RNA gene libraries for tag-sequencing, Illumina libraries were constructed using the primer pair B969F (ACGCGHNRAACCTTACC) and BA1406R (ACGGGCRGTGWGTRCAA) [37] targeting the V6–V8 variable region, and sequenced by paired-end sequencing of 2 × 250 bp read length. Library construction of the 16S rRNA gene and subsequent sequencing were conducted at Novogene Co., Ltd services (Cambridge, UK) on an Illumina Novaseq 6000 platform. Not all samples were sequenced in the same flowcells, but including batch identity in the models did not qualitatively change statistical inferences (i.e., statistical significance; results not shown).

### Sequence analysis

We processed the microbiota sequences in QIIME2 v2023.9 [38]. First, we removed primers using the Cutadapt plugin v4.5 [39], and then reads were quality-filtered and clustered into Amplicon Sequence Variants (ASVs) using DADA2 v1.26.0 [40]. Each ASV was taxonomically classified using the classify-sklearn method against the Silva 138 database clustered at 99% similarity [41], and non-bacterial sequences were removed. To detect and filter out potential contaminations, we used the R package ‘decontam’ v1.22.0 [42, 43], with a threshold of 0.4 (prevalence method), which identified 89 ASVs as contaminants from field and negative controls (extraction blanks). We used the method align-to-tree-mafft-fasttree, based on MAFFT aligner [44] and FastTree [45], to generate a rooted phylogenetic tree of our sequences. Finally, the rooted tree, the ASV and taxonomy tables were imported to R v4.3.2 [43] using the R package ‘phyloseq’ v1.46.0 [46].

### Statistical analyses

#### Interspecific differences in bacterial composition and phylosymbiosis

Because phylosymbiosis requires that bacterial communities differ significantly among host species [16], we first examined interspecific differences in bacterial community composition (beta diversity) and in alpha diversity metrics (observed ASVs, Shannon diversity and Faith phylogenetic diversity), all models and results are presented in ESM S2.1. We rarefied the ASV table at the minimum sampling depth (5,564) and calculated Bray-Curtis (community composition based on ASV relative abundance), unweighted UniFrac (phylogenetic distance based on presence/absence of ASVs), and weighted UniFrac (phylogenetic distance including the relative abundance of ASVs) distance matrices using the function *distance* in the ‘phyloseq’ R package v1.46.0 [46]. PERMANOVAs with 9,999 permutations were first conducted in PRIMER7 v7.0.17 separately for adults and nestlings/juveniles, and since no age-related differences were detected (see ESM Tables S1–S2), we pooled all individuals per species for subsequent analyses. For alpha diversity analyses, we used General Linear Models (GLMs) and Mixed Models (GLMMs). PERMANOVAs, GLMs and GLMMs included species identity as fixed factor, and when multiple samples per nest were available (e.g., siblings and mother-offspring pairs), we included nest identity nested within species as a random factor to account for potential pseudoreplication due to kinship-relatedness. Differences in bacterial community composition across host species were visualised using Principal Coordinates Analysis (PCoA). Furthermore, we explored the predominant genera per species and population by generating a heatmap based on ASV relative abundances aggregated at the genus level, focusing on the 20 most abundant genera in the dataset.

To test for phylosymbiosis, we built a host phylogeny from the majority-rule consensus of 100 phylogenetic trees obtained from http://birdtree.org [47], using the Ericson backbone based on multilocus nuclear DNA [48]. We constructed the host phylogeny at the species level. Furthermore, due to the lack of genetic data for hoopoe subspecies, we built three alternative trees reflecting all possible diversification scenarios of the considered subspecies (for more details, see ESM S2.2). All statistical analyses were performed with information at the species and subspecies levels. To match taxa between the phylogenetic trees and bacterial distance matrices, we generated two ASV tables: one at the species level (seven species) and another at the subspecies level (six species plus three hoopoe subspecies). We estimated distance matrices for the core microbiome of each species and subspecies, which was defined using a standardised approach based on occupancy-abundance patterns and the ranked contribution of ASVs to beta diversity [49]. For the common scimitarbill, represented by a single sample, we selected the 50 most abundant ASVs as a proxy. After identifying the core ASVs for each host taxa, we combined them, yielding 2,884 ASVs for further analysis. Because statistical inferences can be sensitive to the criteria used to define the core microbiome [50], we also conducted complementary analyses using a more restrictive filtering (i.e., ASVs that were present in at least 50% of the samples and with a minimum relative abundance of 0.0001).

We assessed phylosymbiosis using Mantel tests and Procrustes analysis. Two-tailed Mantel tests with 10,000 permutations were performed with the ‘ecodist’ R package v2.1.3 [51] to calculate Pearson correlation coefficients between the host patristic distance matrix and the bacterial beta diversity distance matrix. For the Procrustes analysis [52], both matrices were first converted into coordinate configurations using PCoA. The analyses were performed with the *procrustes* and *protest* functions in ‘vegan’ R package v2.6.10 [53]. For both methods, our primary beta diversity metric was unweighted UniFrac distance since it emphasizes the phylogenetic relationships and presence/absence of bacterial symbionts rather than their relative abundance. Nonetheless, we also tested phylosymbiosis using Bray-Curtis and weighted UniFrac distances under both core microbiome definitions [54]. These supplementary analyses are reported in ESM Sections S2.5 and S2.6. Finally, dendrograms based on unweighted UniFrac distance and host phylogeny were compared using the ‘dendextend’ R package v1.19.0 [55] and visualised with the *tanglegram* function.

#### Interspecific differences in antimicrobial activity and functional phylosymbiosis

We explored interspecific variation in antimicrobial activity using non-parametric tests (Kruskal-Wallis or Wilcoxon rank-sum), adjusting post hoc *p*-values from Kruskal-Wallis tests with the Benjamini-Hochberg method. Nesting adults and nestlings/juveniles were pooled per species, as no age-related differences in antimicrobial activity were detected (see ESM Table S3). After confirming significant differences in antimicrobial activity across species, we tested predictions of functional phylosymbiosis following two complementary approaches (see Fig. S3). First, we explored the association between antimicrobial activity and host phylogeny using Mantel tests with 10,000 permutations. Separate analyses were conducted for: (i) all species and subspecies, (ii) hoopoes and woodhoopoes only, and (iii) hornbill species only. For each model, the dependent variable was the Euclidean distance matrix of antimicrobial activity (using the ‘vegan’ R package v2.6.10 [53]), calculated as the mean values of antimicrobial activity of bacterial communities against each of the three indicator bacteria, averaged at the subspecies or species level. The independent matrix was that of host phylogenetic distances.

Second, we used multiple regression tests on matrices (MRMs) with 10,000 permutations using the ‘ecodist’ R package v2.1.3 [51] to test the association between antimicrobial activity of individuals and their bacterial community beta diversity, after controlling for host phylogenetic and geographic distances. Phylogenetic distances were defined as patristic distances between host taxa, with intraspecific distances set to zero. Geographic distances were computed from nest coordinates (latitude and longitude) using the *distm* function with the Haversine formula from the ‘geosphere’ R package v1.5.20 [56]. The geographic distance matrix controls for possible spatial autocorrelation effects and, since identical coordinates were assigned to individuals from the same nests, it also accounts for potential pseudoreplication due to kinship-relatedness. Initial models included all covariates, but we subsequently removed non-significant predictors (*p* > 0.05) to obtain final reduced models (see ESM Table S6 for models before variable selection). For reasons explained above, in the main text, we present results from the unweighted UniFrac distance matrices of the occupancy-based core microbiome, and the results from the other beta diversity indexes and more restricted core microbiomes are shown in the ESM (Table S5). All models were performed using unstandardised and standardised matrices (i.e., mean-centred and scaled to unit standard deviation, with diagonal values kept at zero). Since the statistical inferences did not differ qualitatively between the two approaches (results not shown), we report here those obtained with standardised matrices.

#### Detecting specific bacterial taxa associated with antimicrobial activity

To identify specific bacteria associated with antimicrobial activity against the three indicator bacteria, we used sparse Partial Least Squares (sPLS) regression models. We focused on species-level models for the African, European, and Asian hoopoes, as well as the Namibian green woodhoopoe, because they showed significant associations between antimicrobial activity and bacterial community composition (see results). Global models (including all hoopoe subspecies and green woodhoopoes, or all hornbill species) and other species-specific models were explored and presented in the ESM (Table S7). To focus on core ASVs, we included only ASVs present in at least 4 samples per species and constituting more than 0.008% of the total ASV counts (the number of ASVs included in each model is presented in Fig. S8). All models included the CLR-transformed ASV relative abundances as predictors (see Fig. S3), and the standardised antimicrobial activity (mean-centred and scaled to unit variance) as the response variable. Model performance was assessed using 5-fold cross-validation with 50 repeats for models with at least 20 samples, and leave-one-out cross-validation otherwise. Only the first latent component was retained (see ESM Fig. S8) as no additional component exceeded the established Q^2^ validation threshold of 0.095 [57]. The European hoopoe model showed Q^2^ values below this threshold; therefore, the results should be interpreted with caution. The sPLS models were run in R v4.3.2 using the *spls* function, and the networks were generated using the *network* function, both from the ‘mixOmics’ R package v6.26.0 [58].

## Results

### Interspecific differences in bacterial composition and phylosymbiosis

**Fig. 1:**
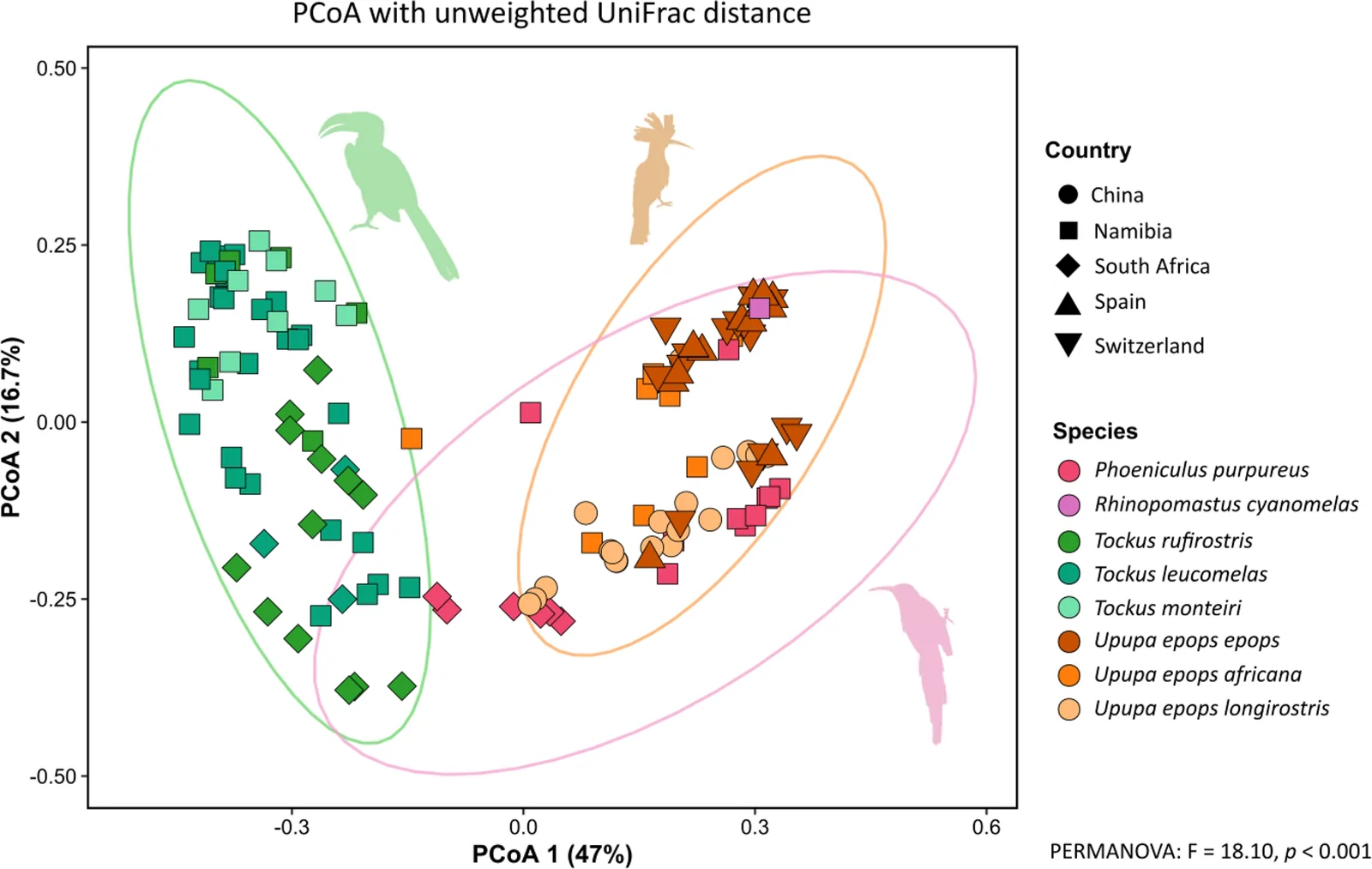
Principal coordinates analysis (PCoA) based on unweighted UniFrac distances of uropygial secretion microbiota. Different species and subspecies are distinguished by colours; hoopoes (orange), woodhoopoes (purple), and hornbills (green), and populations by geometric forms. Ellipses represent 95% confidence intervals for each group. Result of PERMANOVA with 9,999 permutations testing for interspecific differences in uropygial secretion microbiota is also included

**Fig. 2:**
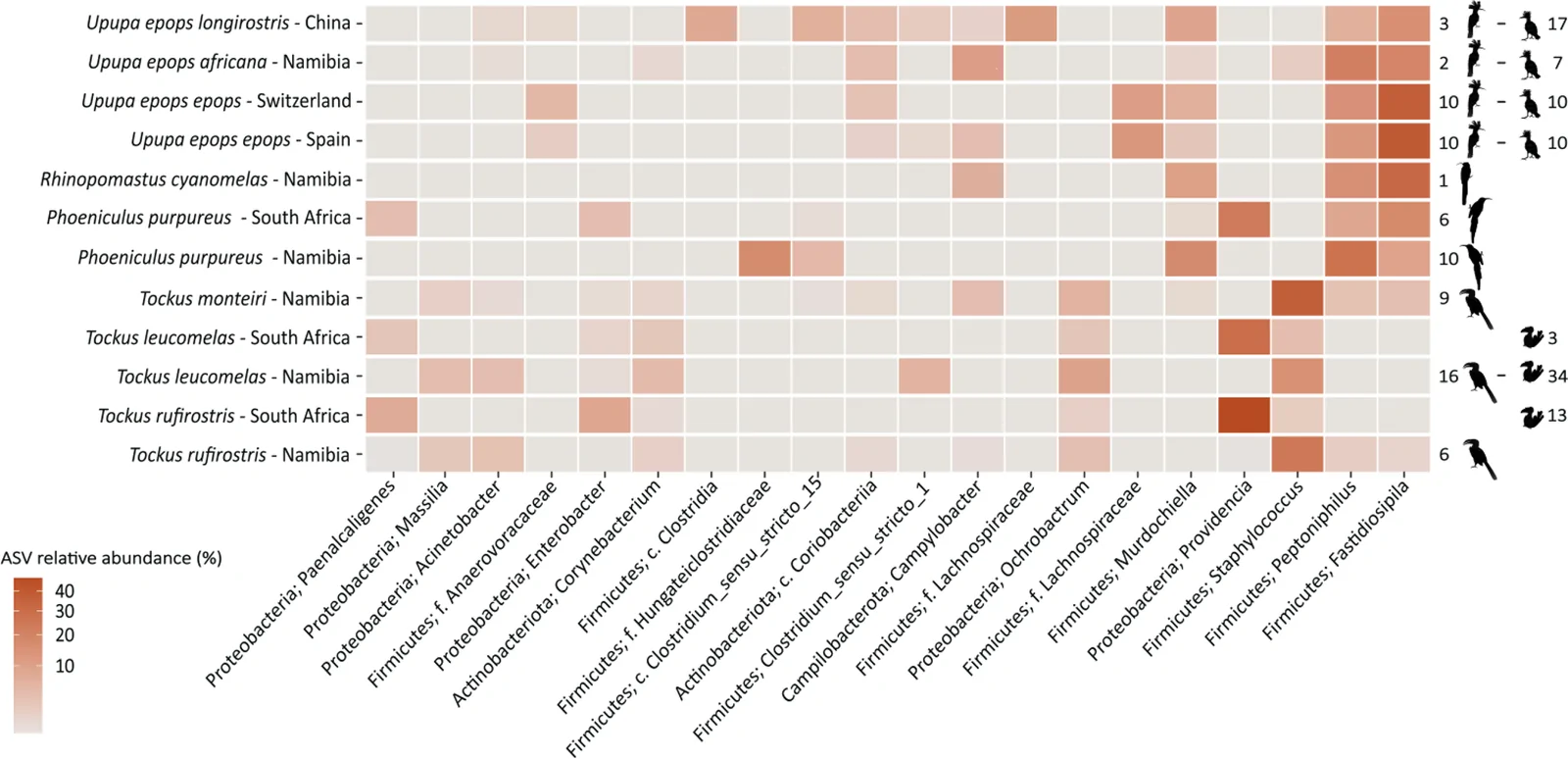
Heatmap showing the 20 most abundant bacterial genera in the uropygial gland secretions of the studied species. Data are grouped by species or subspecies and by country of origin. ASV relative abundances were normalised and aggregated at the genus level. For unclassified taxa, the most specific available taxonomic rank (e.g., family or order) is shown, along with the corresponding phylum. Colour intensity represents relative abundance. Pictograms indicate species identity, age class (analysed together), and sample size. All sampled birds were nesting individuals

We sequenced the uropygial gland secretions of 69 hoopoes (40 European hoopoes, 9 African hoopoes, and 20 Asian hoopoes), 17 woodhoopoes (16 green woodhoopoes and 1 scimitarbill), 59 hornbills (31 yellow-billed hornbills, 9 Monteiro hornbills, and 19 red-billed hornbills), and 24 rollers (Table 1). In total, we obtained 5,934,440 sequences classified into 20,652 ASVs, with an average of 35,115 sequences per sample (SD = 14,591.89). After rarefaction at the minimum sampling depth (5,564 reads), the ASV table contained 15,948 unique ASVs assigned to 31 phyla and 850 genera, with Firmicutes (59.9%) and Proteobacteria (22%) as the most abundant phyla. We found statistically significant interspecific differences across all beta diversity distance matrices (PERMANOVAs, *p* < 0.001) (see ESM Table S2 and Fig. S5). Moreover, interspecies comparisons (e.g., unweighted UniFrac distances, PERMANOVA: F = 18.10, *p* < 0.001) revealed a phylogenetically consistent clustering pattern (Fig. 1). The bacterial community of hoopoes and woodhoopoes was dominated by *Peptoniphilus* and *Fastidiosipila* (Fig. 2), whereas *Staphylococcus* and *Ochrobactrum* were abundant across all *Tockus* species. That result was irrespective of the geographic location of sampled populations (Fig. 2).

**Fig. 3:**
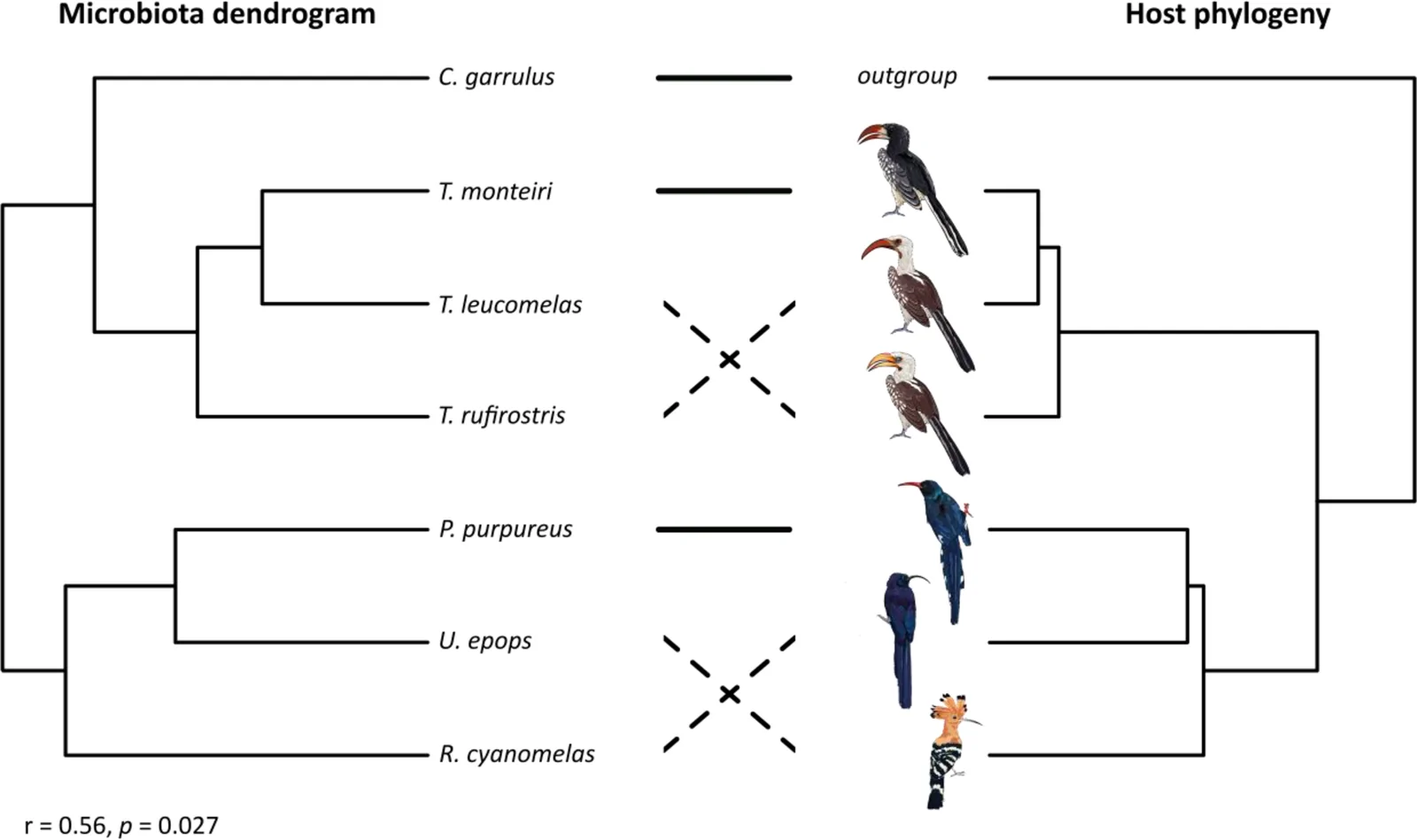
Tanglegram comparing the host phylogeny (right) and bacterial community of the uropygial secretion (left). The host phylogenetic tree reflects evolutionary relationships among species, and the bacterial dendrogram unweighted UniFrac distances. The central lines connect the microbiota and phylogenetic positions of each species, and the crossed-dashed lines indicate discordance between the nodes of the phylogenetic tree and that of the bacterial communities’ dendrogram. Mantel test result (Pearson correlation) between the host phylogenetic distance matrix and the bacterial beta diversity distance matrix is shown

In line with phylosymbiotic predictions, Mantel tests revealed a significant positive correlation between host phylogeny and bacterial community composition at the species (*r* = 0.56, *p* = 0.027; Fig. 3) and subspecies levels (i.e., all three possible divergence scenarios (*r* > 0.60, *p* = 0.001)). Procrustes analyses showed the same pattern, at both the species level (symmetric Procrustes correlation = 0.91, *p* = 0.012; Fig. S7) and the three hoopoe divergence scenarios (symmetric Procrustes correlation > 0.87, *p* for the African hoopoe = 0.001, *p* for the Asian and European hoopoes = 0.002, Fig. S7). Results remained statistically significant when considering the restrictive core microbiome and unweighted UniFrac distance matrices (ESM S2.5). Comparable patterns were also found using Bray-Curtis or weighted UniFrac distances, except in Mantel tests when applying weighted UniFrac distances with the restrictive core microbiome definition (ESM Table S5).

### Interspecific differences in antimicrobial activity and functional phylosymbiosis

**Fig. 4:**
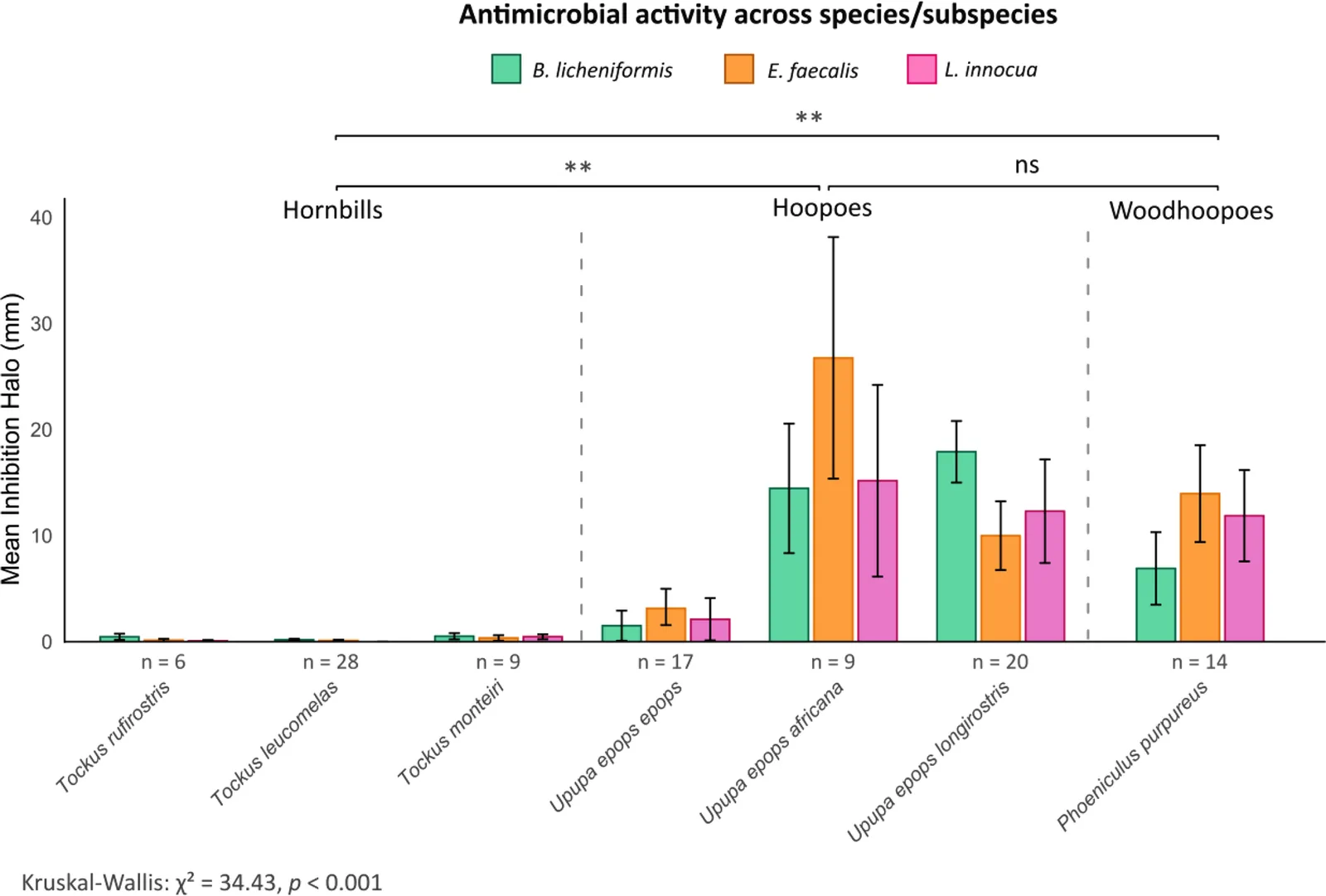
Antimicrobial activity of bacterial communities of the uropygial gland from the studied species. Antimicrobial activity (average ± SE) was estimated as the average mean of the inhibition halos of bacterial isolates multiplied by log-transformed bacterial density and was separately estimated for each of the three indicator bacterial strains used: *Bacillus licheniformis, Listeria innocua,* and *Enterococcus faecalis*. Results from Kruskal-Wallis and post hoc Dunn tests with Benjamini-Hochberg correction performed at species/subspecies and group level (hornbills, woodhoopoes, and hoopoes) are shown. Asterisks denote significance levels (**: *p* < 0.01). Although the sample from the common scimitarbill was also analysed, no bacteria presented antimicrobial activity and is therefore not shown in the figure, but considered for the statistical analyses (value = 0)

We found significant interspecific differences in antimicrobial activity of uropygial secretion bacterial isolates against all three indicator bacteria (Kruskal-Wallis results: *B. licheniformis*: χ^2^ = 35.25, *p* < 0.001; *L. innocua*: χ^2^ = 42.44, *p* < 0.001; *E. faecalis*: χ^2^ = 26.53, *p* < 0.001; see Fig. 4 and Table S4 for post-hoc comparisons). Within the families Upupidae and Phoeniculidae, isolates from Asian and African hoopoes and from the green woodhoopoe, showed the highest levels of antimicrobial activity (Fig. 4). By contrast, isolates from the European hoopoe exhibited reduced level of antagonism in comparison to the other hoopoe subspecies, and the common scimitarbill isolates showed no antimicrobial activity (Fig. 4). The isolates of all hornbill species showed lower level of antimicrobial activity, with some even displaying non-antagonism. Similar patterns were observed when comparing bacterial density; isolates from hoopoes and green woodhoopoes exhibited markedly higher densities than hornbills (see ESM S2.3, Fig. S6).

When considering the isolates of all studied bird taxa, antimicrobial activity was positively, though not significantly associated with phylogenetic distances. That was the case independently of assuming that African hoopoe (*r* = 0.23; *p* = 0.089), Asian hoopoe (*r* = 0.20; *p* = 0.122), or European hoopoe diverged first (*r* = 0.23; *p* = 0.083). In the model restricted to hoopoes and woodhoopoes, the association turned negative and non-significant across all three subspecies divergence scenarios (i.e., diverging first the African hoopoe (*r* = −0.22; *p* = 0.608), the Asian hoopoe (*r* = −0.39; *p* = 0.296), or the European hoopoe (*r* = −0.16; *p* = 0.701). The model focused exclusively on hornbill species also showed a non-significant association between phylogeny and antimicrobial activity (*r* = 0.02; *p* = 1).

Beta diversity of bacterial communities was significantly negatively associated with antimicrobial activity when considering all sampled individuals. That was the case independently of the assumed pattern of divergence among hoopoe subspecies (i.e., diverging first the African hoopoe (β = −0.21, *p* = 0.009), the Asian hoopoe (β = −0.21, *p* = 0.011), or European hoopoe (β = −0.20, *p* = 0.015)), and after controlling for the significant effects of host phylogeny (African hoopoe β = 0.25; Asian hoopoe β = 0.25; European hoopoe β = 0.23; *p* < 0.001 in all cases). By contrast, when restricting the dataset to hoopoes and woodhoopoes, the relationship became positive (β = 0.16, *p* = 0.042 for the three divergence scenarios), and in this case, the effect of host phylogeny was not significant and removed from the final models (see ESM Table S6). In hornbills, beta diversity was also negatively associated with antimicrobial activity, though not statistically significant (β = −0.22, *p* = 0.089), after controlling for a significant phylogenetic effect (β = 0.23, *p* = 0.014). Analyses restricted to a more stringent core microbiome and based on unweighted UniFrac distance matrices yielded statistically significant associations depending on the hoopoe divergence scenario in models including all sampled individuals. In contrast, the models including information of all individuals but based on Bray-Curtis or weighted UniFrac distance matrices did not yield significant associations. Focusing on hoopoes and woodhoopoes, models based on Bray-Curtis distances were significant for both definitions of core microbiome. However, when weighted UniFrac was used as the explanatory variable, no significant associations were found. In hornbills, none of the models yielded statistically significant associations regardless of the core microbiome definition or beta diversity metric used (Bray-Curtis or weighted UniFrac) (see ESM Table S5).

### Specific bacterial taxa associated with antimicrobial activity in hoopoes and woodhoopoes

**Fig. 5:**
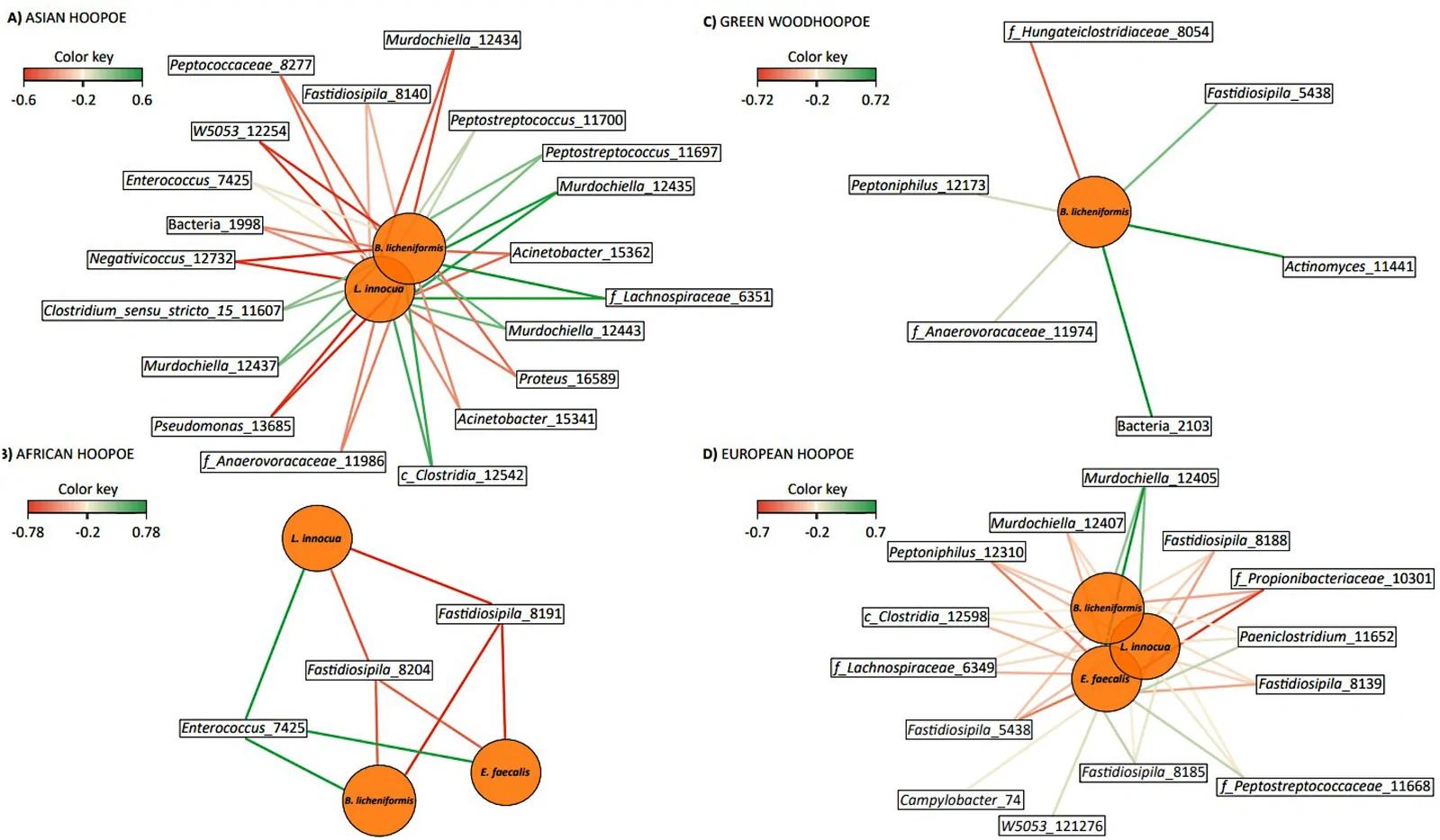
Network representation of sPLS models. The networks show associations between ASVs and antimicrobial activity against three indicator bacteria (*Bacillus licheniformis, Listeria innocua,* and *Enterococcus faecalis*) retained in the first latent component. Green edges indicate positive correlations, while orange edges indicate negative correlations. The colour intensity reflects the strength of the correlation coefficient, with darker shades indicating stronger correlations. ASVs are classified at the genus level when possible, otherwise, classification is indicated by *f* for family level, *c* for class level, and bacteria when no more specific taxonomic classification was available

Identity of the bacterial strains that in sPLS models appeared closely linked with the antimicrobial activities of isolates from hoopoes and green woodhoopoes varied depending on the considered bird species or subspecies and the indicator bacteria used in antagonistic tests (see correlation coefficients in ESM Table S7). Despite these context-dependent differences, ASVs from the same bacterial genera were consistently associated with antimicrobial activity of isolates against one, two, or all three indicator bacteria across host taxa. Highlighting the importance of these key ASVs, we found that the relative abundance of an *Enterococcus* (ASV 7425) was positively associated with antimicrobial activity of the bacterial communities of the African (against the three indicator bacteria) and Asian hoopoes (against *B. licheniformis* and *L. innocua*) (Fig. 5). Although ASV 7425 was not selected in the sPLS models performed with information from the rest of species, it did appear when considering the microbiome of the uropygial secretion of the European hoopoe, but not in the green woodhoopoe. ASVs from the genus *Fastidiosipila* were generally negatively associated with the estimated antimicrobial activity of isolates in different species. However, there were cases where the same ASV correlated positively for one species but negatively for another. For example, ASV 5438 was negatively correlated with antimicrobial activity against the three indicator bacteria in the European hoopoe, but positively correlated against *B. licheniformis* in the green woodhoopoe (Fig. 5). *Peptoniphilus* ASVs were also associated positively with antimicrobial activity against *B. licheniformis* (ASV 12,173) or negatively associated against the three indicator bacteria (ASV 12,310), depending on the host species. Other bacterial strains, such as ASVs from the genus *Murdochiella*, were recurrently positive correlates (ASVs 12,435, 12,443, 12,405), while *Negativicoccus* (ASV 12,732) and *Pseudomonas* (ASV 13,685) showed a negative association with antimicrobial activity against two or all three indicator bacteria. Detailed associations between the relative abundance of ASVs and the antimicrobial activity of isolates against indicator bacteria retained in the first latent component are presented in Fig. 5.

A diagram showing an overview of the performed models and the statistical significance of each analysis is presented in ESM Figure S3.

## Discussion

Our study of eight Bucerotiformes taxa demonstrated consistent interspecific differences in the composition and diversity of the uropygial gland microbiome and showed that closely related bird species harboured more similar bacterial communities than would be expected by chance. We also found consistent positive associations between bacterial composition and antimicrobial activity of hoopoe and woodhoopoe isolates, suggesting that bacteria conferring immunity to hosts may influence the observed pattern of phylosymbiosis. Finally, we identified bacteria that associated with antimicrobial properties of symbionts isolated from the uropygial gland secretion. Below, we discuss the importance and novelty of these findings highlighting potential evolutionary relationships between hosts and their uropygial gland microbial community.

Phylosymbiosis has been mainly explored in bacterial communities of the gut and is frequently observed across the animal phylogeny, with the exception of flying vertebrates (i.e., birds and bats) [59–61]. Physiological constraints imposed by flight on intestine mass, such as reduced gut length and shorter retention time, may explain the absence of phylosymbiosis in those taxa [62, 63]. This explanation implies that patterns of phylosymbiosis may still emerge when focusing on bacterial communities from other host organs not directly constrained by flight. The uropygial gland represents a relatively stable and enclosed environment shaped by birds’ anatomical and physiological traits. This protected environment influences the composition and diversity of the symbiotic bacteria and enables hosts to promote the establishment of particular taxa [64]. Antimicrobial compounds in uropygial secretion are partially derived from bacterial symbiont metabolism [23, 35, 65] and vary across bird species [66]. Thus, because the morphological and physiological characteristics of the uropygial gland have a phylogenetic component [66], and likely determine antimicrobial properties (some of them mediated by bacterial symbionts), the detected pattern of phylosymbiosis could be the result of evolutionary processes selecting for particular antimicrobial defences. Our results provide mixed support for that possibility. On the one hand, there was no association between the antimicrobial activity of the bacterial communities of Bucerotiformes and the host phylogenetic distances. The limited number of taxa considered, which prevents detecting a statistically significant effect for the estimated effect size, might explain the lack of association. Moreover, the evolutionary history of these species might not reflect their risk of infection by pathogens. In accordance, phylogenetically distant birds often face similar ecological selection pressures, as suggested by the weak phylogenetic signal of the interspecific association between density of potential pathogenic bacteria in avian nests and the immune responses (Pagel λ = 0) [67]. Beyond host phylogeny and antimicrobial properties of the symbiotic microbiome, several alternative mechanisms may contribute to the observed phylosymbiosis pattern. For instance, ecological and behavioural factors, such as habitat characteristics, social interactions, diet, and geographic proximity, can influence microbial exposure and colonisation independently of phylogenetic relationships [17]. In our study, phylogenetically distant species coexist in the same geographic location (all hornbill species, woodhoopoes, and African hoopoes), whereas phylogenetically closer subspecies are separated by continents. This pattern suggests that geographic distance or environmental dissimilarities are unlikely to be determining the detected phylogenetic signal. On the other hand, several behavioural (e.g., sociality, vertical transmission of symbionts [68]) or physiological (e.g., immunity, hormonal state [69–71]) host traits influence the composition of symbiotic microbial community, and, thus, could also explain the detected phylosymbiosis pattern. Exploring the associations between host immunity and antimicrobial properties of symbionts would shed light on the importance of the hosts’ immune system in influencing the evolution of their microbial community.

Even though antimicrobial defence did not follow the host phylogeny, the antimicrobial properties of symbiotic microorganisms [23, 72], or host immunity [73–75], may still have evolved rapidly in response to host species-specific infection risks. As a result, even when phylosymbiosis is observed, the functional benefits provided by bacterial symbionts may depend more on their specific assembly than on their hosts’ phylogenetic history [23]. The strength of the association between the bacterial symbiont composition and antimicrobial activity largely depended on the species considered in the analyses. The variation in antimicrobial activity among species may be explained by interspecific differences in the evolved antiparasitic strategies (i.e., immune, behavioural, or symbiotic mediated defences). For instance, hoopoes and green woodhoopoes have developed a specialised relationship with antibiotic-producing bacteria harboured in their uropygial gland, which directly contributes to their antimicrobial defence [24–26, 35]. In contrast, hornbills might rely on alternative defence mechanisms that do not primarily involve symbiotic bacteria (e.g., the use of substances like millipede secretions [76–78]), that would also protect the nest environment against pathogens. As predicted by the hypothesis of functional phylosymbiosis, we found a positive association when considering the three hoopoe subspecies and woodhoopoes, but not when considering all Bucerotiformes taxa or only hornbills. Unlike hornbills, hoopoes and woodhoopoes produce dark, malodorous secretions harbouring a very high bacterial density (except the common scimitarbill, see ESM S2.3). The antimicrobial properties of these bacteria have mainly been studied in European hoopoes, concluding that some strains produce broad-spectrum antimicrobial substances [31, 79], and that the enterococci strains with broader antimicrobial spectrum are the most prevalent [80]. Taken together, these previous findings suggest that European hoopoes are able to select symbiotic strains with the highest antimicrobial effects. A recently published article [81] provides a detailed explanation of the particularities of the uropygial gland of hoopoes that could facilitate the selection of their specific core microbiome. In the European hoopoe, bacteria living within the gland are confined in the papilla by means of a specialised keratinised corneous layer that compartmentalises a cavity for symbiont cultivation. Within the papilla, the bird provides substances and physical-niches through the disaggregation of keratin laminae and secretions from the glandular tissue, which may be used to target specific bacteria. Additionally, there is vertical transmission from mother to offspring of some core symbionts like *Peptoniphilus*, explaining its high prevalence and specificity. Our estimates of the antimicrobial activity of isolates from green woodhoopoes, Asian and African hoopoes were even stronger than those observed in European hoopoes. Then, given that the antagonism of these species and subspecies varied and associated positively with their bacterial composition, it is likely that these species, as the European hoopoe, evolve a gland environment that favours the growth of these particular bacteria. Supporting this possibility, sPLS models highlighted specific bacterial taxa that best explain the antimicrobial activity of isolates against particular indicator bacteria. Interestingly, the strength and direction of these associations varied depending on the indicator strain, the host, and the ASV identity. Although most bacterial taxa differed across hosts, four genera consistently appeared in multiple species or subspecies. ASVs from three of them, *Fastidiosipila*, *Murdochiella,* and *Peptoniphilus,* were either positively or negatively associated with antimicrobial activity depending on the host identity. These genera are Gram-positive anaerobic fermenters commonly found in glandular or mucosal microbiota and in animal guts [82, 83]. Because our antagonistic tests were performed in aerobiosis, and most species within these genera are obligate anaerobes, it is unlikely that the isolates used in these tests belonged to these genera. However, since some strains are aerotolerant (e.g., *Fastidiosipila* has been shown to grow under 2–6% oxygen [84]), we cannot discard that some of the isolates belonged to those taxa. Thus, the most probable explanation is that the observed associations reflect indirect effects of these taxa on broader community structure and function [85, 86]. Moreover, the observed host differences in antimicrobial activity of their symbionts could reflect risk of pathogenic infection, and favour certain host traits acting as selective filters for key bacteria shaping microbial network, selecting more antagonistic communities under high-risk conditions [87]. In European hoopoes, ASVs from *Murdochiella, Fastidiosipila*, and *Peptoniphilus* are core members of the uropygial secretion. Notably, some evidence suggests that *Peptoniphilus* ASVs are vertically transmitted from mother to offspring, and are negatively correlated with *Enterococcus*, the genus primarily responsible for antimicrobial production in this system [81]. This finding suggests a competitive or regulatory interaction where *Peptoniphilus* (and potentially other core members) may modulate the abundance or activity of primary antimicrobial producers like *Enterococcus*, thereby indirectly shaping the overall antimicrobial capacity of the community. Future work should aim to clarify the role of specific strains by genetically characterising isolates used in antagonism assays and experimentally inoculating hosts with bacteria identified by sPLS analyses. In addition, identifying the specific pathogens against which these strains act would help to understand the ecological pressures driving the maintenance and evolution of this symbiotic relationship.

Interestingly, the above-mentioned three key genera are almost exclusively found in the Upupidae and Phoeniculidae families (Fig. 2). *Enterococcus* was another genus associated with antimicrobial activity in these families. Previous studies have shown that enterococci provide most benefits in the European hoopoe [65, 88, 89]. Our results indicate that the same ASV belonging to *Enterococcus* associated positively with antimicrobial activity in Asian (see also Soler et al. [35]) and African hoopoes. With few exceptions, different hosts harboured different strains of these four genera, consistent with a possible coevolutionary process underlining the detected functional phylosymbiosis. However, our statistical approach cannot resolve causality or distinguish true ecological interactions from other non-random patterns [90, 91]. Therefore, the mechanistic effects of such bacteria on the entire community should be tested experimentally to reach firm conclusions about their ecological roles within the community. To move beyond correlative inferences, future studies should focus on experimental manipulations to directly test whether the introduction of these strains increases overall community-level antagonism or alters the abundance and inhibition capacity of specific taxa.

## Electronic supplementary material

Below is the link to the electronic supplementary material.

Supplementary material 1

## Data Availability

The raw 16S rRNA gene sequences for hoopoes, woodhoopoes, and hornbills were deposited in GenBank under accession number PRJNA1345256, and for rollers (outgroup) under the accession number PRJNA1248315. Associated metadata including antimicrobial activity values, ASV tables, taxonomy tables, phylogenetic trees, and code are available in CSIC Institutional Repository (DIGITAL.CSIC: https://doi.org/10.20350/digitalCSIC/22662).
