# Supplementary material for: Functional phylosymbiosis in birds: phylogeny and antimicrobial properties of bacterial symbionts in Bucerotiformes
Source: Anim Microbiome. 2026 Aug 7;8:116. doi: 10.1186/s42523-026-00608-y (PMC13741672; doi:10.1186/s42523-026-00608-y)
Supplement: Supplementary file 1 [file 42523_2026_608_MOESM1_ESM.docx]

**Supplementary Material:**

# Functional phylosymbiosis in birds: Phylogeny and antimicrobial properties of bacterial symbionts in Bucerotiformes

**SECTION 1: Additional Methods**

**S1.1 Sampling locations and fieldwork**

The two populations of European hoopoe were sampled: one in the Hoya de Guadix, a high plateau in southern Spain (37°24′08″N, 3°04′04″W) between April and July 2021, and another in Valais, southwestern Switzerland (46°10′N, 7°12′E), in 2014. At the Spanish location, we also sampled European rollers during the 2021 breeding season. Both species were sampled from reproduction events in artificial nest boxes, and breeding activity was identified using a portable endoscope. The African hoopoe was sampled in Otjiwarongo, Namibia, at the Cheetah Conservation Fund farms (20°29′14″S, 17°02′03″E) between November and December 2022. For African hoopoes, we installed nest boxes following the measurements of those used in the Spanish population (35 cm height x 18 cm width x 21 cm depth, 24 cm from the bottom to hole entrance, and 5.5 cm hole diameter), but they did not use them and preferred natural holes (i.e., tree holes, a ground-level metal-covered cavity, and a hole of a non-active termite mound). The Asian hoopoe was sampled in the Wenchang countryside, Hainan Island, southern China (19°40′N, 110°45′E) between May and July 2023. Although appropriate artificial nest boxes were also available in this population, they preferred to breed in natural cavities built by rodents or in holes in the walls of buildings on local farms. In holes with narrow entrances, we carefully open the nest entrance and, after sampling, the removed material was glued back leaving the nest entrance or nest wall in its original shape (see Fig. S1). From the green woodhoopoe we sampled two populations: one from Wiese and Aris farms near Windhoek, Namibia, between September and February 2022-2023, and another one in Mogalakwena, South Africa (22°43′S, 28°46′E). The common scimitarbill was sampled at the Cheetah Conservation Fund in Namibia in November 2023. Green woodhoopoes and common scimitarbills were captured using mist nets placed at the entrances of natural cavities. The southern yellow-billed hornbill was sampled between November and January 2021-2022 at the Cheetah Conservation Fund and in February 2023 in Mogalakwena. The southern red-billed hornbill was sampled in both the Cheetah Conservation Fund and Mogalakwena during the 2023 breeding season. Monteiro's hornbill was sampled at the Cheetah Conservation Fund in 2023. All hornbill nests were located in artificial nest boxes, and active broods were identified by observing the nest entrance sealed. None of the sampled nests were abandoned after the procedure.

**Figure S1. Opening of a natural cavity for sampling.** (A) An example of how a natural cavity was carefully opened to access the nestlings. (B) The removed pieces were glued back to their original position. (C) The nestlings inside the cavity before the pieces were reattached.

**S1.2 Interspecific variation in uropygial glands and secretions**

Among hornbill species, the glands were similar in appearance and size. They had narrow gland lobes, lacked a visible papilla with accumulated secretion, and were often surrounded by a dense tuft of feathers at the opening (Fig. S2). In contrast, green woodhoopoes, hooopes, and scimitarbills had prominent glands with two well-defined wide lobes. The papilla full of secretion is also apparent in these species. The uropygial secretion of hornbills, hoopoes, scimitarbills, and green woodhoopoes differed in colour and odour. In hornbills, the secretion was odourless and the colour was beige/yellowish, whereas in hoopoes and woodhoopoes, the colour was dark brown and the odour was strong and malodorous. In hoopoes and scimitarbills, brooding females and nestlings exclusively produce the dark, malodorous secretion during the breeding season, while non-brooding females and males secrete white, odourless secretion [1]. In contrast, the green woodhoopoe maintains the association with bacteria and the special characteristics throughout the year, with no sex-specific differences [2, 3].

**Figure S2. Uropygial gland morphology and secretion collection.** (A) Uropygial gland of a Yellow-billed hornbill and collection of uropygial secretion from a Monteiro’s female using a microcapillary. Both pictures were taken at the Cheetah Conservation Fund, Namibia. (B) Uropygial gland of a green woodhoopoe, showing a secretion-filled papilla. The uropygial secretion was collected using a sterilised micropipette tip during sampling at Aris Farm, Namibia (photos by Dirk Heinrich). (C) Uropygial glands of hoopoes, the first image shows a European hoopoe with a secretion-filled papilla and a dark-brown drop emerging. The following two images show an African hoopoe female’s gland after secretion collection, and the sampling process using a micropipette tip. The European hoopoe was sampled in Guadix, Spain, and the African hoopoe at the Cheetah Conservation Fund, Namibia.

**S1.3 Additional details on antimicrobial activity estimation**

As described in the main text, we estimated individual-level antimicrobial activity by testing the antimicrobial activity of five colonies using the double-layer technique against three indicator bacteria (*B. licheniformis*, *L. innocua*, and *E. faecalis*). Briefly, each isolated strain was replicated by spotting onto three TSA Petri dishes (30-35 isolates per plate) and incubated at 37 °C for 24 h for antimicrobial production. Subsequently, each plate was overlaid with 6 ml of soft agar (Brain Heart Infusion added 0.8% agar, Scharlau Chemie S.A., Barcelona) inoculated with 100 μl of an overnight liquid culture of the indicator strain at 37 °C. After incubating the plates overnight at 37 °C, the antimicrobial activity of each isolated colony was measured with a digital calliper as the width of the growth-inhibition halos around the spot of isolates. Then, we estimated individual-level antimicrobial activity by averaging the inhibition halos produced by up to five bacterial colonies per individual against each of the three indicator strains. These mean values were multiplied by log-transformed bacterial density of the sample to obtain values of antimicrobial activity of the entire bacterial community. In some samples, no bacterial colonies grew on the plates (i.e., CFU = 0); in these cases, we assigned an antagonism value of zero for each indicator strain. In other cases, some colonies showed expansive, swarming-like growth patterns, hampering the estimation of their antimicrobial activity. In these cases, we assumed the mean inhibition value of all measurable colonies from the same host species against the corresponding indicator strain. This occurred in 19 colonies: 2 colonies from the Namibian green woodhoopoe, 7 from the Asian hoopoe, and 10 from the Monteiro’s hornbill. This substitution allowed us to retain individuals in the dataset while minimizing bias associated with missing or irregular measurements.

**SECTION 2: Extended Statistical Analysis and Results**

Given the number of statistical models performed to test our predictions, we provide a schematic diagram to summarize the analytical approach and the significance of the main results.

**Figure S3.** Diagram summarizing the performed main analyses: (1) interspecific differences in alpha and beta diversity, (2) phylosymbiosis, (3) interspecific differences in antimicrobial activity (antimicrobial activity x bacterial density), (4) functional phylosymbiosis, and (5) sPLS regressions. Beta diversity was calculated using unweighted UniFrac for the main analyses, while results using Bray-Curtis and weighted UniFrac distances are provided in the ESM Table S5. All analyses were repeated using two previously defined core microbiome definitions. For additional sPLS analyses, see ESM Table S7 and Fig. S8. Checkmarks (✓) and crosses (✗) indicate statistically significant and non-significant results, respectively. Sample sizes represent the number of individuals or species/subspecies (average values) included in each analysis. For phylosymbiosis and functional phylosymbiosis, values indicate the number of ASVs used in beta diversity calculations (based on occupancy-abundance patterns and ranked ASV contributions to beta diversity). In sPLS models, "ASVs" refers to predictor ASVs retained after filtering steps (present in ≥ 4 samples and > 0.008% of total ASV counts). See Main text Table 1 for more detailed sample sizes and information on which individuals per species were also tested for antimicrobial activity.

**S2.1 Interspecific differences in alpha and beta diversity**

We tested for interspecific differences in the microbiome in terms of alpha and beta diversities. To account for possible differences in sampling and sequencing success, we rarefied the ASV table 100 times to the minimum sampling depth (5,564) [4]. From these 100 rarefied tables, we calculated the average values for three alpha diversity indices: richness (observed ASVs), Shannon diversity, and Faith’s phylogenetic diversity. Richness and Shannon’s diversity were calculated using the alpha function in the 'microbiome' R package v1.24.0 [5], and Faith's phylogenetic diversity was calculated using the 'picante' R package v1.8.2 [6]. For certain species, we sampled adult and nestling/juvenile individuals, therefore, we first ran separate models for adults and nestlings/juveniles and, after confirming no age differences, we combined information from these two ages for subsequent analyses (hereafter, combined models). For models including only adults, species identity was used as a categorical fixed factor. In models focused on nestlings and juveniles, species identity was also treated as a fixed factor, while nest identity nested within species was included as a random factor since some nests contained more than one sampled nestling. The combined models included species as a fixed factor and nest identity nested within species to account for multiple samples from the same nest (i.e., siblings or mother-offspring pairs). We used General Linear Models (GLMs) and Mixed Models (GLMMs) to test for differences in each alpha diversity index. To explore interspecific differences in beta diversity, we rarefied the ASV table at the minimum sampling depth and calculated Bray-Curtis, unweighted UniFrac, and weighted UniFrac distance matrices. Beta diversity models followed the same structure as alpha diversity models. We used PERMANOVAs with 9,999 permutations to assess differences in beta diversity. GLMs and GLMMs were performed using TIBCO Statistica software v14 [7], and PERMANOVAs were conducted using PRIMER7 v7.0.17 (PRIMER-e).

We observed significant interspecific differences in both alpha (GLMMs *p* < 0.001) and beta (PERMANOVAs *p* < 0.001) diversity across all model types (see Tables S1-S2 and Fig. S4-S5).

**Table S1.** Results of general linear models (GLMs) and general linear mixed models (GLMMs) exploring the effect of species on bacterial diversity at the ASV level. (A) Models include only adult individuals: *Tockus monteiri* (n = 9), *Tockus leucomelas* (n = 11), *Tockus rufirostris* (n = 6), *Upupa epops* *epops* (n = 20), *Upupa epops africana* (n = 2), *Upupa epops longirostris* (n = 3), *Phoeniculus purpureus* (n = 12), and *Rhinopomastus cyanomelas* (n = 1). (B) Only nestlings (*R. cyanomelas* and *T. monteiri* were excluded due to the absence of nestling data): *T. leucomelas* (n = 20), *T. rufirostris* (n = 13), *U. e. epops* (n = 20), *U. e. africana* (n = 7), *U. e. longirostris* (n = 17), and *P. purpureus* (n = 4), or (C) Combined models. When multiple individuals were sampled per nest, nest ID was included as a random factor (models B and C). *P*-values < 0.05 are shown in bold.

|  |  | **ALPHA DIVERSITY** | | | | | | | | |
| --- | --- | --- | --- | --- | --- | --- | --- | --- | --- | --- |
|  |  | **Richness** | | | **Shannon** | | | **PD** | | |
|  |  | F | df | *p* | F | df | *p* | F | df | *p* |
| **(A) All species (adults)** | |  |  |  |  |  |  |  |  |  |
| Species | | 18.60 | 7,56 | **<0.001** | 16.56 | 7,56 | **<0.001** | 38.87 | 7,56 | **<0.001** |
| **(B) All species (nestlings/juveniles)** | |  |  |  |  |  |  |  |  |  |
| Species  Nest ID (Species) | | 8.76 | 5,40.98 | **<0.001** | 18.90 | 5,19.68 | **<0.001** | 11.38 | 5,38.69 | **<0.001** |
|  |  | 3.54 | 48,27 | **<0.001** | 0.83 | 48,27 | 0.724 | 2.68 | 48,27 | **0.004** |
| **(C) All species (combined)** | |  |  |  |  |  |  |  |  |  |
| Species  Nest ID (Species) | | 15.14 | 7,76.05 | **<0.001** | 13.20 | 7,74.60 | **<0.001** | 21.25 | 7,74.89 | **<0.001** |
|  |  | 2.23 | 69,68 | **0.001** | 2.81 | 69,68 | **<0.001** | 2.67 | 69,68 | **<0.001** |

**Figure S4.** Weighted means (± 95% confidence intervals) of the three alpha diversity metrics, Richness (observed ASVs), Shannon diversity, and Faith’s phylogenetic diversity (PD) in the uropygial secretion microbiota across the studied species. Hornbill species are shown in shades of green colours, woodhoopoes in purple-pink, and hoopoes in orange. Sample sizes per species are also shown.

**Table S2.** PERMANOVA results based on 9,999 permutations testing for differences in uropygial secretion bacterial composition (beta diversity) across bird species at the ASV level. We show results for (A) Adults: *Tockus monteiri* (n = 9), *Tockus leucomelas* (n = 11), *Tockus rufirostris* (n = 6), *Upupa epops* *epops* (n = 20), *Upupa epops africana* (n = 2), *Upupa epops longirostris* (n = 3), *Phoeniculus purpureus* (n = 12), and *Rhinopomastus cyanomelas* (n = 1), and (B) Nestlings: *T. leucomelas* (n = 20), *T. rufirostris* (n = 13), *U. e. epops* (n = 20), *U. e. africana* (n = 7), *U. e. longirostris* (n = 17), and *P. purpureus* (n = 4)) or (C) Combined information of adults and nestlings. Nest ID was included as a random factor in models B and C, since we included multiple individuals sampled from the same nests (siblings or mother-offspring). Statistically significant p-values (p < 0.05) are shown in bold.

|  |  | **BETA DIVERSITY** | | | | | | | | |
| --- | --- | --- | --- | --- | --- | --- | --- | --- | --- | --- |
|  |  | **Bray-Curtis** | | | **unweighted UniFrac** | | | **weighted UniFrac** | | |
|  |  | Pseudo-F | df | *p* | Pseudo-F | df | *p* | Pseudo-F | df | *p* |
| **(A) All species (adults)** | |  |  |  |  |  |  |  |  |  |
| Species | | 4.85 | 7,56 | **<0.001** | 16.11 | 7,56 | **<0.001** | 12.75 | 7,56 | **<0.001** |
| **(B) All species (nestlings/juveniles)** | |  |  |  |  |  |  |  |  |  |
| Species ID  Nest ID (Species ID) | | 5.69 | 5.3,48 | **<0.001** | 11.82 | 5.19,48 | **<0.001** | 26.97 | 5.08,48 | **<0.001** |
|  |  | 1.53 | 48,27 | **<0.001** | 1.17 | 48,27 | 0.128 | 1.18 | 48,27 | 0.210 |
| **(C) All species (combined)** | |  |  |  |  |  |  |  |  |  |
| Species ID  Nest ID (Species ID) | | 6.34 | 7,75.91 | **<0.001** | 18.10 | 7,78.64 | **<0.001** | 19.52 | 7,74.89 | **<0.001** |
|  |  | 2.27 | 69,68 | **<0.001** | 1.62 | 69,68 | **<0.001** | 2.67 | 69,68 | **<0.001** |

**Figure S5.** Principal Coordinates Analysis (PCoA) based on Bray-Curtis (left) and weighted UniFrac (right) distances of uropygial secretion microbiota. Colours indicate the sampled species, while shapes denote the country of origin. Hoopoe subspecies/populations are shown in shades of orange, woodhoopoes in purple-pink, and hornbills in green. Ellipses represent 95% confidence intervals for each group: hoopoes (orange), woodhoopoes (purple), and hornbills (green).

**S2.2 Host phylogeny**

For the host phylogeny, we created a majority-rule consensus tree derived from 100 phylogenetic trees obtained from [http://birdtree.org](http://birdtree.org/) [8]. *Tockus rufirostris* is one of the five species in which *Tockus erythrorhynchus* (red-billed hornbills’ complex, see Kemp A*, et al.* [9], Gonzalez JC*, et al.* [10]) has recently split, and thus, to build a phylogenetic tree, we used information of *Tockus erythrorhynchus*, which is the only species of the red-billed hornbills’ complex with information in birdtree. The European roller, a species from the order Coraciiformes (a sister clade of Bucerotiformes), was used as an outgroup to root the phylogenetic trees of interest, and the consensus tree was estimated using Mesquite software [11]. Genetic information was unavailable for the three hoopoe subspecies (European, African, and Asian). To incorporate these subspecies in the analyses, we constructed three phylogenetic trees that reflect the three possible scenarios of hoopoe diversification. We assumed equal branch length among nodes reflecting the diversification of hoopoes, while maintaining the distance to the node where *Upupa epops* diversified constant and equal to the phylogenetic tree that does not consider hoopoe subspecies.

**S2.3 Interspecific differences in bacterial density**

We assessed interspecific differences in bacterial density across all sampled species and subspecies. For hoopoes and woodhoopoes, density was estimated as colony-forming units per millilitre (CFU/mL). For hornbills, because their samples were not serially diluted, we used total colony counts per plate. All estimates were log-transformed to approach a normal distribution. Non-parametric Kruskal-Wallis test showed significant differences in bacterial density among species, with hoopoes and green woodhoopoes showing higher bacterial loads than hornbills (Fig. S6). The common scimitarbill was an exception, as its uropygial secretion had very low bacterial density, and its antimicrobial activity was zero. Since we only had one sample of this species, we cannot discard a technical issue during the plating procedure, as the secretion’s characteristics (dark and malodorous) matched those of other hoopoes and green woodhoopoes. Overall, these density differences were similar to those found in antimicrobial activity, where species with higher bacterial densities also tended to show stronger antimicrobial activity.

**Figure S6.** Differences in log-transformed bacterial counts across all species and subspecies. Kruskal-Wallis test showed significant differences among species, and post hoc comparisons were conducted using Dunn’s test with Benjamini-Hochberg correction. Density values equal to zero represent samples in which no bacterial growth was observed. Only significant pairwise comparisons are shown (*p*.adjust < 0.05), indicated by asterisks: *p* < 0.05: *, *p* < 0.01: **, *p* < 0.001: ***.

**S2.4 Interspecific age-related differences in antimicrobial activity and post-hoc results**

We first examined whether antimicrobial activity differed between adults and nestlings/juveniles within species (Table S3) and, since no significant age-related differences were observed, information from individuals of different age classes was pooled together. Comparisons of antimicrobial activity between species are presented in the main text.

**Table S3.** Results of non-parametric Wilcoxon tests assessing age-related differences in antagonism estimates against three indicator strains. Only individuals with measured antimicrobial activity are included in the models (see Table 1 main text). Yellow-billed hornbill included only individuals sampled in Namibia, and green woodhoopoes included individuals sampled in Namibia and South Africa.

|  | *B. licheniformis* | *L. innocua* | *E. faecalis* |
| --- | --- | --- | --- |
| **European hoopoe** |  |  |  |
| Females vs. nestlings | W = 33; *p* = 0.794 | W = 42; *p* = 0.257 | W = 32; *p* = 0.743 |
| **Asian hoopoe** |  |  |  |
| Females vs. nestlings | W = 20.5; *p* = 0.633 | W = 21.5; *p* = 0.710 | W = 26.5; *p* = 0.956 |
| **African hoopoe** |  |  |  |
| Females vs. nestlings | W = 6; *p* = 0.873 | W = 5.5; *p* = 0.760 | W = 6.5; *p* = 1 |
| **Green woodhoopoe** |  |  |  |
| Adults vs. juveniles | W = 22; *p* = 0.805 | W = 25; *p* = 0.497 | W = 28.5; *p* = 0.239 |
| **Yellow-billed hornbill** |  |  |  |
| Females vs. nestlings | W = 85; *p* = 0.536 | - | W = 110.5; *p* = 0.082 |

|  | *B. licheniformis* | *L. innocua* | *E. faecalis* |
| --- | --- | --- | --- |
| **All species** |  |  |  |
| *P. purpureus – R. cyanomelas* | Z = 0.73; *p* = 0.762 | Z = 1.12; *p* = 0.430 | Z = 1.23; *p* = 0.407 |
| *P. purpureus – T. rufirostris* | Z = 0.52; *p* = 0.770 | Z = 1.9; p = 0.160 | Z = 2.11; *p* = 0.121 |
| *R. cyanomelas – T. rufirostris* | Z = -0.47; *p* = 0.745 | Z = -0.22; *p* = 0.927 | Z = -0.23; *p* = 0.920 |
| *P. purpureus – T. leucomelas* | Z = 1.67; *p* = 0.295 | Z = 3.55; *p* = **0.004** | Z = 3.56; *p* = **0.005** |
| *R. cyanomelas – T. leucomelas* | Z = -0.21; *p* = 0.898 | Z = 0; *p* = 1 | Z = -0.11; *p* = 0.915 |
| *T. rufirostris – T. leucomelas* | Z = 0.65; *p* = 0.756 | Z = 0.52; *p* = 0.801 | Z = 0.3; *p* = 0.930 |
| *P. purpureus – T. monteiri* | Z = 0.57; *p* = 0.762 | Z = 1.55; *p* = 0.262 | Z = 1.49; *p* = 0.346 |
| *R. cyanomelas – T. monteiri* | Z = -0.49; *p* = 0.758 | Z = -0.48; *p* = 0.772 | Z = -0.61; *p* = 0.803 |
| *T. rufirostris – T. monteiri* | Z = -0.02; *p* = 0.984 | Z = -0.51; *p* = 0.781 | Z = -0.75; *p* = 0.708 |
| *T. leucomelas – T. monteiri* | Z = -0.8; *p* = 0.795 | Z = -1.31; *p* = 0.356 | Z = -1.38; *p* = 0.360 |
| *P. purpureus – U. epops* | Z = 1.48; *p* = 0.352 | Z = 2.54; *p* = **0.044** | Z = 2.2; *p* = 0.155 |
| *R. cyanomelas – U. epops* | Z = -0.22; *p* = 0.926 | Z = -0.24; *p* = 0.947 | Z = -0.47; *p* = 0.854 |
| *T. rufirostris – U. epops* | Z = 0.6; *p* = 0.773 | Z = -0.02; *p* = 1 | Z = -0.5; *p* = 0.867 |
| *T. leucomelas – U. epops* | Z = -0.04; *p* = 1 | Z = -0.80; *p* = 0.626 | Z = -1.21; *p* = 0.398 |
| *T. monteiri – U. epops* | Z = 0.71; *p* = 0.741 | Z = 0.62; *p* = 0.749 | Z = 0.38; *p* = 0.893 |
| *P. purpureus – U. epops africana* | Z = -0.77; *p* = 0.772 | Z = -0.21; *p* = 0.898 | Z = -0.18; *p* = 0.923 |
| *R. cyanomelas – U. epops africana* | Z = -1.03; *p* = 0.603 | Z = -1.19; *p* = 0.411 | Z = -1.28; *p* = 0.399 |
| *T. rufirostris – U. epops africana* | Z = -1.1; *p* = 0.581 | Z = -1.93; *p* = 0.167 | Z = -2.10; *p* = 0.111 |
| *T. leucomelas – U. epops africana* | Z = -2.29; *p* = 0.104 | Z = -3.27; *p* = **0.008** | Z = -3.24; *p* = **0.011** |
| *T. monteiri – U. epops africana* | Z = -1.21; *p* = 0.528 | Z = -1.59; *p* = 0.283 | Z = -1.51; *p* = 0.364 |
| *U. epops – U. epops africana* | Z = -2.1; *p* = 0.144 | Z = -2.44; *p* = 0.051 | Z = -2.11; *p* = 0.138 |
| *P. purpureus – U. epops longirostris* | Z = -2.86; *p* = **0.029** | Z = -1.35; *p* = 0.356 | Z = 0.12; *p* = 0.941 |
| *R. cyanomelas – U. epops longirostris* | Z = -1.72; *p* = 0.302 | Z = -1.59; *p* = 0.259 | Z = -1.2; *p* = 0.376 |
| *T. rufirostris – U. epops longirostris* | Z = -2.68; *p* = **0.041** | Z = -3; *p* = **0.015** | Z = -2.13; *p* = 0.156 |
| *T. leucomelas – U. epops longirostris* | Z = -5.27; *p* < **0.001** | Z = -5.57; *p* < **0.001** | Z = -3.84; *p* = **0.003** |
| *T. monteiri – U. epops longirostris* | Z = -3.09; *p* = **0.019** | Z = -2.82; *p* = **0.023** | Z = -1.49; *p* = 0.321 |
| *U. epops – U. epops longirostris* | Z = -4.64; *p* < **0.001** | Z = -4.2; *p* < **0.001** | Z = -2.29; *p* = 0.156 |
| *U. epops africana – U. epops longirostris* | Z = -1.66; *p* = 0.269 | Z = -0.95; *p* = 0.535 | Z = 0.29; *p* = 0.898 |

**Table S4.** Post hoc Dunn tests with Benjamini-Hochberg correction for multiple comparisons.
Z indicates the Dunn test statistic, and p refers to the Benjamini-Hochberg adjusted p-value.

**S2.5 Phylosymbiosis and Functional phylosymbiosis – unweighted UniFrac using restrictive core microbiome**

To further test phylosymbiosis and functional phylosymbiosis (explored as the association between antimicrobial activity and bacterial beta diversity), we repeated the analyses presented in the main text using a more restrictive condition for core microbiome. This approach included only ASVs present in at least 50% of the samples and with a minimum relative abundance of 0.0001. All main analyses were based on unweighted UniFrac distance matrices. As described in the main text, we assessed phylosymbiosis using Mantel tests and Procrustes Analyses, and functional phylosymbiosis through multiple regression on distance matrices (MRMs) with 10,000 permutations. MRMs included host phylogenetic and geographic distances as explanatory variables. However, since geographic distance did not significantly explain variation in antimicrobial activity (*p* > 0.05 in all models), it was excluded from the final models. Using this core microbiome, we still observed strong support for phylosymbiosis. At the species level, host phylogeny significantly correlated with bacterial community composition (Mantel r = 0.61, *p* = 0.011; symmetric Procrustes correlation = 0.91, *p* = 0.012). This pattern held across all three alternative divergence scenarios of hoopoes. Mantel tests: r = 0.68, *p* < 0.001. Procrustes analyses for the three hoopoe divergence scenarios: symmetric Procrustes correlation for the African and European hoopoes = 0.89, and for the Asian hoopoe = 0.88, all *p* < 0.001.

In terms of functional phylosymbiosis, when analysing all species together, the results varied depending on the assumed divergence scenario. Under the African-first scenario, bacterial beta diversity significantly predicted antimicrobial activity (β = -0.16, *p* = 0.038). The association was significant when assuming the Asian hoopoe diverged first (β = -0.15, *p* = 0.046), but marginally non-significant when the European subspecies diverged first (β = -0.14, *p* = 0.060). In contrast, host phylogenetic distance consistently explained a significant proportion of the variation in antimicrobial activity across all three divergence scenarios, with similar effect sizes (African hoopoe: β = 0.22, Asian hoopoe: β = 0.21, European hoopoe: β = 0.20, all *p* = 0.001). In the model restricted to hoopoes and woodhoopoes, we found a non-significant but positive association between bacterial beta diversity and antimicrobial activity across the three divergence scenarios (β = 0.13; *p* = 0.111). In this model, only bacterial beta diversity was included as an explanatory variable, as host phylogenetic distance had a non-significant effect. In hornbills, the association between beta diversity and antagonism was far from statistical significance (β = -0.08; *p* = 0.545), but we found a statistically significant effect of phylogenetic distance on antimicrobial activity (β = 0.20; *p* = 0.031).

**S2.6 Phylosymbiosis and Functional phylosymbiosis – Alternative beta diversity metrics using both definitions of core microbiome**

Following the recommendations discussed in [12], we repeated the analyses using alternative beta diversity metrics: Bray-Curtis dissimilarity and weighted UniFrac distances. For both phylosymbiosis and functional phylosymbiosis, we applied the same modelling structure described in the main text, using both core microbiome definitions. Results for all combinations of metrics and core definitions are presented in Table S5.

**Table S5.** Results of phylosymbiosis (Mantel tests and Procrustes Analyses) and functional phylosymbiosis (MRMs) using Bray-Curtis and weighted UniFrac distance matrices, under two core microbiome definitions. Core microbiome 1 corresponds to the main definition used in the manuscript, based on occupancy–abundance and the ranked contribution of ASVs to beta diversity. **Core microbiome 2** included ASVs present in ≥ 50% of samples and with a relative abundance ≥ 0.0001. Mantel tests, Procrustes Analyses, and MRMs followed the same procedures as the corresponding analyses in the main text using unweighted UniFrac distance matrices. In the MRM model restricted to hoopoes and woodhoopoes, host phylogenetic distance is not included because it was not significant and was removed from the final model. Statistically significant associations (p < 0.05) are shown in bold.

| **Analysis** | **Model** | **Independent variable** | ***Core microbiome 1*** | ***Core microbiome 2*** |
| --- | --- | --- | --- | --- |
| **Phylosymbiosis**  **(Mantel tests)** | **(A) Species level** | Bray-Curtis | r = 0.70, *p* = **0.015** | r = 0.69, *p* = **0.016** |
|  |  | weighted UniFrac | r = 0.57, *p* = **0.020** | r = 0.38, *p* = 0.111 |
|  | **(B) Subspecies:** |  |  |  |
|  | African diverged first | Bray-Curtis | r = 0.70, *p* = **0.001** | r = 0.69, *p* = **0.001** |
|  |  | weighted UniFrac | r = 0.60, *p* = **0.003** | r = 0.45, *p* = 0.053 |
|  | Asian diverged first | Bray-Curtis | r = 0.70, *p* = **0.001** | r = 0.70, *p* = **0.001** |
|  |  | weighted UniFrac | r = 0.59, *p* = **0.005** | r = 0.44, *p* = 0.063 |
|  | European diverged first | Bray-Curtis | r = 0.70, *p* = **0.001** | r = 0.69, *p* = **0.001** |
|  |  | weighted UniFrac | r = 0.59, *p* = **0.004** | r = 0.45 *p* = 0.058 |
| **Phylosymbiosis**  **(Procrustes analyses)** | **(A) Species level** | Bray-Curtis | r = 0.86, *p* = **0.013** | r = 0.87, *p* = **0.013** |
|  |  | weighted UniFrac | r = 0.87, *p* = **0.020** | r = 0.82, *p* = **0.027** |
|  | **(B) Subspecies:** |  |  |  |
|  | African diverged first | Bray-Curtis | r = 0.87, p < **0.001** | r = 0.88, p < **0.001** |
|  |  | weighted UniFrac | r = 0.86, *p* = **0.001** | r = 0.81, *p* = **0.004** |
|  | Asian diverged first | Bray-Curtis | r = 0.87, p < **0.001** | r = 0.88, p < **0.001** |
|  |  | weighted UniFrac | r = 0.85, *p* = **0.002** | r = 0.80, *p* = **0.004** |
|  | European diverged first | Bray-Curtis | r = 0.87, p < **0.001** | r = 0.88, p < **0.001** |
|  |  | weighted UniFrac | r = 0.85, *p* = **0.002** | r = 0.81, *p* = **0.001** |
| **Functional phylosymbiosis**  **(MRMs)** | **(A) All species:** |  |  |  |
|  | African diverged first | Bray-Curtis | β = -0.01, *p* = 0.895 | β = 0.02, *p* = 0.768 |
|  |  | Host phylogeny | β = 0.12, *p* = **0.001** | β = 0.10, *p* = **0.005** |
|  |  | weighted UniFrac | β = -0.11, *p* = 0.184 | β = -0.00, *p* = 0.979 |
|  |  | Host phylogeny | β = 0.18, *p* = **0.006** | β = 0.12, *p* = **0.024** |
|  | Asian diverged first | Bray-Curtis | β = -0.01, *p* = 0.870 | β = 0.02, *p* = 0.800 |
|  |  | Host phylogeny | β = 0.12, *p* = **0.001** | β = 0.11, *p* = **0.006** |
|  |  | weighted UniFrac | β = -0.10, *p* = 0.215 | β = 0.00, *p* = 0.980 |
|  |  | Host phylogeny | β = 0.17, *p* = **0.006** | β = 0.11, *p* = **0.022** |
|  | European diverged first | Bray-Curtis | β = 0.00, *p* = 0.999 | β = 0.03, *p* = 0.673 |
|  |  | Host phylogeny | β = 0.11, *p* = **0.004** | β = 0.09, *p* = **0.018** |
|  |  | weighted UniFrac | β = -0.09, *p* = 0.246 | β = 0.01, *p* = 0.940 |
|  |  | Host phylogeny | β = 0.16, *p* = **0.008** | β = 0.10, *p* = **0.032** |
|  | **(B) Only hoopoes and woodhoopoes** |  |  |  |
|  | African diverged first | Bray-Curtis | β = 0.17, *p* = **0.005** | β = 0.16, *p* = **0.008** |
|  |  | weighted UniFrac | β = 0.08, *p* = 0.427 | β = 0.11, *p* = 0.213 |
|  | Asian diverged first | Bray-Curtis | β = 0.17, *p* = **0.005** | β = 0.16, *p* = **0.008** |
|  |  | weighted UniFrac | β = 0.08, *p* = 0.427 | β = 0.11, *p* = 0.213 |
|  | European diverged first | Bray-Curtis | β = 0.17, *p* = **0.005** | β = 0.16, *p* = **0.008** |
|  |  | weighted UniFrac | β = 0.08, *p* = 0.427 | β = 0.11, *p* = 0.213 |
|  | **(C) Only hornbills** | Bray-Curtis | β = -0.32, *p* = 0.096 | β = -0.21, *p* = 0.182 |
|  |  | Host phylogeny | β = 0.22, *p* = **0.018** | β = 0.22, *p* = **0.019** |
|  |  | weighted UniFrac | β = -0.05, *p* = 0.654 | β = -0.06, *p* = 0.643 |
|  |  | Host phylogeny | β = 0.19, *p* = **0.023** | β = 0.19, *p* = **0.025** |

**Figure S7.** Procrustean superimposition for PCoA-scaled host phylogeny distances and bacterial beta diversity based on a core microbiome of 2884 ASVs (unweighted UniFrac, weighted UniFrac, and Bray-Curtis). Circles represent the host phylogeny and diamonds the bacterial community. (A) Analysis at the species level. (B) Analysis including the three hoopoe subspecies, with the African hoopoe as the first divergent lineage. The symmetric Procrustes correlation (r) is shown for all models.

**S2.7 Functional phylosymbiosis – Full models**

To allow reproducibility, we report here the results of the initial MRM models testing the association between antimicrobial activity and bacterial beta diversity, including host phylogenetic and geographic distances as covariates. Phylogenetic distances were calculated as patristic distances among host species or subspecies, while geographic distances were computed from nest coordinates (latitude and longitude). All models were run using 10,000 permutations. Table S6 showed the results of full models before variable selection.

**Table S6.** Results from full multiple regression on distance matrices (MRMs) models testing the association between antimicrobial activity and bacterial beta diversity, with host phylogenetic distance and geographic distance included as covariates. Results are provided for all three beta diversity metrics (unweighted UniFrac, Bray-Curtis, and weighted UniFrac) and both definitions of the core microbiome. Non-significant predictors (*p* > 0.05) were excluded from the final models presented in the main text.

| **Model** | **Indep. variable** | ***Core microbiome 1*** | ***Core microbiome 2*** |
| --- | --- | --- | --- |
| **(A) All species:** |  |  |  |
| African diverged first | unweighted UniFrac | β = -0.19, *p* **= 0.022** | β = -0.14, *p* **=** 0.076 |
|  | Host phylogeny | β = 0.21, *p* **= 0.006** | β = 0.17, *p* **= 0.020** |
|  | Geography | β = 0.09, *p* **=** 0.248 | β = 0.10, *p* **=** 0.186 |
|  | Bray-Curtis | β = -0.02, *p* **=** 0.732 | β = -0.00, *p* **=** 0.984 |
|  | Host phylogeny | β = 0.08, *p* **= 0.037** | β = 0.07, *p* **= 0.072** |
|  | Geography | β = 0.12, *p* **=** 0.112 | β = 0.12, *p* **=** 0.118 |
|  | weighted UniFrac | β = -0.08, *p* **=** 0.308 | β = 0.02, *p* = 0.796 |
|  | Host phylogeny | β = 0.13, *p* **=** 0.068 | β = 0.06, *p* = 0.330 |
|  | Geography | β = 0.10, *p* **=** 0.167 | β = 0.12, *p* = 0.097 |
| Asian diverged first | unweighted UniFrac | β = -0.18, *p* **= 0.025** | β = -0.13, *p* **=** 0.094 |
|  | Host phylogeny | β = 0.20, *p* **= 0.006** | β = 0.16, *p* **= 0.024** |
|  | Geography | β = 0.08, *p* **=** 0.303 | β = 0.09, *p* **=** 0.214 |
|  | Bray-Curtis | β = -0.02, *p* **=** 0.792 | β = 0.01, *p* **=** 0.935 |
|  | Host phylogeny | β = 0.08, *p* **= 0.048** | β = 0.06, *p* **=** 0.096 |
|  | Geography | β = 0.12, *p* **=** 0.118 | β = 0.12, *p* **=** 0.123 |
|  | weighted UniFrac | β = -0.07, *p* **=** 0.359 | β = 0.03, *p* = 0.741 |
|  | Host phylogeny | β = 0.11, *p* **=** 0.079 | β = 0.05, *p* = 0.367 |
|  | Geography | β = 0.10, *p* **=** 0.174 | β = 0.12, *p* **=** 0.099 |
| European diverged first | unweighted UniFrac | β = -0.17, *p* **= 0.035** | β = -0.12, *p* **=** 0.116 |
|  | Host phylogeny | β = 0.18, *p* **= 0.012** | β = 0.14, *p* **= 0.036** |
|  | Geography | β = 0.09, *p* **=** 0.230 | β = 0.10, *p* **=** 0.165 |
|  | Bray-Curtis | β = -0.01, *p* **=** 0.867 | β = 0.01, *p* **=** 0.858 |
|  | Host phylogeny | β = 0.07, *p* **=** 0.091 | β = 0.05, *p* **=** 0.174 |
|  | Geography | β = 0.12, *p* **=** 0.104 | β = 0.12, *p* **=** 0.110 |
|  | weighted UniFrac | β = -0.07, *p* **=** 0.401 | β = 0.03, *p* **=** 0.709 |
|  | Host phylogeny | β = 0.10, *p* **=** 0.106 | β = 0.05, *p* **=** 0.433 |
|  | Geography | β = 0.11, *p* **=** 0.141 | β = 0.12, *p* **=** 0.084 |
| **(B) Only hoopoes and woodhoopoes** |  |  |  |
| African diverged first | unweighted UniFrac | β = 0.15, *p* **=** 0.077 | β = 0.12, *p* = 0.190 |
|  | Host phylogeny | β = 0.03, *p* = 0.723 | β = 0.04, *p* = 0.650 |
|  | Geography | β = -0.00, *p* = 0.968 | β = 0.00, *p* = 0.963 |
|  | Bray-Curtis | β = 0.21 *p* = **0.026** | β = 0.20, *p* = **0.033** |
|  | Host phylogeny | β = -0.03, *p* = 0.786 | β = -0.03, *p* = 0.765 |
|  | Geography | β = -0.03, *p* = 0.705 | β = -0.03, *p* = 0.705 |
|  | weighted UniFrac | β = 0.06, *p* = 0.590 | β = 0.10, *p* = 0.302 |
|  | Host phylogeny | β = 0.05, *p* = 0.596 | β = 0.05, *p* = 0.651 |
|  | Geography | β = 0.02, *p* = 0.760 | β = 0.02, *p* = 0.714 |
| Asian diverged first | unweighted UniFrac | β = 0.16, *p* **=** 0.063 | β = 0.13, *p* = 0.150 |
|  | Host phylogeny | β = -0.04, *p* = 0.719 | β = -0.01, *p* = 0.913 |
|  | Geography | β = 0.03, *p* = 0.702 | β = 0.03, *p* = 0.727 |
|  | Bray-Curtis | β = 0.28, *p* = **0.009** | β = 0.27, *p* = **0.012** |
|  | Host phylogeny | β = -0.13, *p* = 0.222 | β = -0.14, *p* = 0.197 |
|  | Geography | β = 0.01, *p* = 0.897 | β = 0.01, *p* = 0.902 |
|  | weighted UniFrac | β = 0.07, *p* = 0.479 | β = 0.11, *p* = 0.231 |
|  | Host phylogeny | β = 0.00, *p* = 0.982 | β = 0.00, *p* = 0.990 |
|  | Geography | β = 0.04, *p* = 0.572 | β = 0.05, *p* = 0.567 |
| European diverged first | unweighted UniFrac | β = 0.16, *p* = 0.059 | β = 0.13, *p* = 0.140 |
|  | Host phylogeny | β = -0.05, *p* = 0.599 | β = -0.03, *p* = 0.746 |
|  | Geography | β = 0.03, *p* = 0.620 | β = 0.04, *p* = 0.593 |
|  | Bray-Curtis | β = 0.30, *p* = **0.007** | β = 0.29, *p* = **0.010** |
|  | Host phylogeny | β = -0.15, *p* = 0.147 | β = -0.16, *p* = 0.128 |
|  | Geography | β = 0.00, *p* = 0.958 | β = 0.00, *p* = 0.981 |
|  | weighted UniFrac | β = 0.08, *p* = 0.447 | β = 0.11, *p* = 0.218 |
|  | Host phylogeny | β = -0.02, *p* = 0.803 | β = -0.02, *p* = 0.808 |
|  | Geography | β = 0.06, *p* = 0.393 | β = 0.06, *p* = 0.389 |
| **(C) Only hornbills** | unweighted UniFrac | β = -0.20, *p* = 0.113 | β = -0.10, *p* = 0.474 |
|  | Host phylogeny | β = 0.24, *p* = **0.011** | β = 0.22, *p* = **0.021** |
|  | Geography | β = -0.08, *p* = 0.466 | β = -0.11, *p* = 0.213 |
|  | Bray-Curtis | β = -0.32, *p* = 0.100 | β = -0.22, *p* = 0.161 |
|  | Host phylogeny | β = 0.24, *p* = **0.014** | β = 0.24, *p* = **0.014** |
|  | Geography | β = -0.10, *p* = 0.228 | β = -0.11, *p* = 0.205 |
|  | weighted UniFrac | β = -0.05, *p* = 0.687 | β = -0.06, *p* = 0.629 |
|  | Host phylogeny | β = 0.21, *p* = **0.017** | β = 0.20, *p* = **0.019** |
|  | Geography | β = -0.10, *p* = 0.242 | β = -0.10, *p* = 0.223 |

**S2.8 sPLS modelling details**

We first performed a sparse partial least squares (sPLS) regression including the three hoopoe subspecies and the Namibian green woodhoopoe population. South African green woodhoopoes and scimitarbills were excluded due to limited sample sizes. After confirming that Q^2^ value exceeded the recommended threshold (Fig. S8), we proceeded to repeat the analysis separately for each hoopoe subspecies and green woodhoopoes. Although models testing for functional phylosymbiosis were not statistically significant for hornbills, we applied the same sPLS approach to explore whether specific ASVs were associated with antimicrobial activity. These analyses included both a global model and species-specific models. The correlations between the ASVs and antagonism variables retained in the first latent component of each model are presented in Table S7. Q^2^ values for each model, together with sample sizes of host species and the number of ASVs used as predictors, are shown in Figure S8. All models included the antimicrobial activity against *B. licheniformis*, *L. innocua* and *E. faecalis*) as response variables.

**Table S7.** Correlation coefficients for each sPLS model. Only ASVs and antagonism variables (corrected by bacterial density) retained in the first latent component are included. The “Species” column refers to the species included in each sPLS model, with “hoopoes and woodhoopoes” model pooling all individuals from the respective species-specific analyses, while “hornbills” model included all hornbill individuals sampled in Namibia. “ASV classification” indicates the best available taxonomic assignment for each ASV. Empty cells indicate that the corresponding antagonism variable was not selected in that component.

| **Species** | **ASV classification** | ***B. licheniformis*** | ***L. innocua*** | ***E. faecalis*** |
| --- | --- | --- | --- | --- |
| Hoopoes_woodhoopoes | *Lachnospiraceae*_6351 | 0.55 | - | - |
| Hoopoes_woodhoopoes | *Murdochiella*_12435 | 0.55 | - | - |
| Hoopoes_woodhoopoes | *Fastidiosipila*_5417 | -0.53 | - | - |
| Hoopoes_woodhoopoes | *Lachnospiraceae*_6349 | -0.53 | - | - |
| Hoopoes_woodhoopoes | *Murdochiella*_12437 | 0.52 | - | - |
| Hoopoes_woodhoopoes | *Varibaculum*_11454 | -0.50 | - | - |
| Hoopoes_woodhoopoes | *Clostridia*_12539 | 0.47 | - | - |
| Hoopoes_woodhoopoes | *Coriobacteriales*_2013 | -0.46 | - | - |
| Hoopoes_woodhoopoes | *Bacteria*_1998 | 0.44 | - | - |
| Hoopoes_woodhoopoes | *Clostridium_sensu_stricto_15*_11606 | 0.44 | - | - |
| Hoopoes_woodhoopoes | *Fastidiosipila*_8139 | -0.43 | - | - |
| Hoopoes_woodhoopoes | *Clostridia*_12542 | 0.43 | - | - |
| Hoopoes_woodhoopoes | *Murdochiella*_12443 | 0.43 | - | - |
| Hoopoes_woodhoopoes | *Murdochiella*_12444 | 0.42 | - | - |
| Hoopoes_woodhoopoes | *Peptoniphilus*_12310 | -0.42 | - | - |
| Hoopoes_woodhoopoes | *Coriobacteriales*_2108 | 0.42 | - | - |
| Hoopoes_woodhoopoes | *Clostridium_sensu_stricto_15*_11607 | 0.41 | - | - |
| Hoopoes_woodhoopoes | *Peptoniphilus*_12312 | 0.41 | - | - |
| Hoopoes_woodhoopoes | *Bacteria*_2002 | -0.38 | - | - |
| Hoopoes_woodhoopoes | *Murdochiella*_12393 | -0.37 | - | - |
| Hoopoes_woodhoopoes | *Campylobacter*_74 | -0.37 | - | - |
| Hoopoes_woodhoopoes | *Enterococcus*_7425 | 0.36 | - | - |
| Hoopoes_woodhoopoes | *Murdochiella*_12429 | 0.36 | - | - |
| Hoopoes_woodhoopoes | *Peptostreptococcales-Tissierellales*_12399 | -0.35 | - | - |
| Hoopoes_woodhoopoes | *Helcococcus*_12457 | -0.35 | - | - |
| Hoopoes_woodhoopoes | *Clostridia*_12558 | -0.33 | - | - |
| Hoopoes_woodhoopoes | *Firmicutes*_2005 | -0.33 | - | - |
| Hoopoes_woodhoopoes | *Peptostreptococcus*_11697 | 0.33 | - | - |
| Hoopoes_woodhoopoes | *Peptostreptococcus*_11700 | 0.32 | - | - |
| Hoopoes_woodhoopoes | *Fastidiosipila*_8185 | -0.32 | - | - |
| African hoopoe | *Enterococcus*_7425 | 0.76 | 0.82 | 0.80 |
| African hoopoe | *Fastidiosipila*_8191 | -0.85 | -0.74 | -0.76 |
| African hoopoe | *Fastidiosipila*_8204 | -0.45 | -0.54 | -0.51 |
| Asian hoopoe | *Pseudomonas*_13685 | -0.61 | -0.56 | - |
| Asian hoopoe | *Murdochiella*_12443 | 0.53 | 0.60 | - |
| Asian hoopoe | *Negativicoccus*_12732 | -0.50 | -0.50 | - |
| Asian hoopoe | *W5053*_12254 | -0.55 | -0.45 | - |
| Asian hoopoe | *Lachnospiraceae*_6351 | 0.50 | 0.49 | - |
| Asian hoopoe | *Proteus*_16589 | -0.52 | -0.46 | - |
| Asian hoopoe | *Murdochiella*_12437 | 0.45 | 0.45 | - |
| Asian hoopoe | *Murdochiella*_12435 | 0.55 | 0.34 | - |
| Asian hoopoe | *Murdochiella*_12434 | -0.54 | -0.35 | - |
| Asian hoopoe | *Acinetobacter*_15362 | -0.40 | -0.49 | - |
| Asian hoopoe | *Clostridia*_12542 | 0.39 | 0.48 | - |
| Asian hoopoe | *Fastidiosipila*_8140 | -0.45 | -0.41 | - |
| Asian hoopoe | *Acinetobacter*_15341 | -0.37 | -0.47 | - |
| Asian hoopoe | *Anaerovoracaceae*_11986 | -0.40 | -0.41 | - |
| Asian hoopoe | *Peptostreptococcus*_11697 | 0.31 | 0.49 | - |
| Asian hoopoe | *Peptostreptococcus*_11700 | 0.28 | 0.44 | - |
| Asian hoopoe | *Bacteria*_1998 | -0.37 | -0.31 | - |
| Asian hoopoe | *Clostridium_sensu_stricto_15*_11607 | 0.34 | 0.32 | - |
| Asian hoopoe | *Peptococcaceae*_8277 | -0.36 | -0.29 | - |
| Asian hoopoe | *Enterococcus*_7425 | 0.29 | 0.36 | - |
| European hoopoe | *Peptoniphilus*_12310 | -0.03 | -0.85 | -0.57 |
| European hoopoe | *Murdochiella*_12405 | 0.38 | 0.35 | 0.58 |
| European hoopoe | *Propionibacteriaceae*_10301 | -0.44 | -0.35 | -0.55 |
| European hoopoe | *Fastidiosipila*_5438 | -0.31 | -0.28 | -0.44 |
| European hoopoe | *Lachnospiraceae*_6349 | -0.38 | 0.01 | -0.48 |
| European hoopoe | *Fastidiosipila*_8185 | 0.18 | 0.26 | 0.42 |
| European hoopoe | *Clostridia*_12598 | -0.26 | -0.23 | -0.36 |
| European hoopoe | *Murdochiella*_12407 | -0.22 | -0.24 | -0.34 |
| European hoopoe | *Paeniclostridium*_11652 | 0.40 | -0.20 | 0.45 |
| European hoopoe | *Fastidiosipila*_8188 | -0.25 | -0.18 | -0.29 |
| European hoopoe | *Murdochiella*_12393 | -0.31 | -0.28 | -0.16 |
| European hoopoe | *Campylobacter*_74 | 0.08 | 0.19 | 0.33 |
| European hoopoe | *Peptoniphilus*_12171 | 0.12 | 0.20 | 0.28 |
| European hoopoe | *Fastidiosipila*_8139 | -0.42 | 0.10 | -0.29 |
| European hoopoe | *Peptostreptococcaceae*_11668 | 0.34 | -0.20 | 0.40 |
| European hoopoe | *Peptostreptococcales-Tissierellales*_12399 | 0.07 | 0.36 | 0.10 |
| European hoopoe | *W5053*_12276 | 0.17 | 0.16 | 0.13 |
| Green woodhoopoe | *Bacteria*_2103 | 0.73 | - | - |
| Green woodhoopoe | *Actinomyces*_11441 | 0.61 | - | - |
| Green woodhoopoe | *Hungateiclostridiaceae*_8054 | -0.57 | - | - |
| Green woodhoopoe | *Anaerovoracaceae*_11974 | 0.48 | - | - |
| Green woodhoopoe | *Fastidiosipila*_5438 | 0.37 | - | - |
| Green woodhoopoe | *Peptoniphilus*_12173 | 0.29 | - | - |
| Hornbills | *Massilia*_14701 | - | 0.57 | - |
| Hornbills | *Massilia*_14571 | - | 0.56 | - |
| Hornbills | *Sphingomonas*_17317 | - | 0.56 | - |
| Hornbills | *Sphingomonas*_17320 | - | 0.55 | - |
| Hornbills | Methylobacterium-Methylorubrum_15769 | - | 0.53 | - |
| Yellow-billed hornbill | *Leucobacter*_9614 | - | - | 0.84 |
| Yellow-billed hornbill | *Sporosarcina*_7076 | - | - | 0.65 |
| Yellow-billed hornbill | *JG30-KF-CM45*_2584 | - | - | 0.64 |
| Yellow-billed hornbill | *Microbacteriaceae*_9409 | - | - | 0.60 |
| Yellow-billed hornbill | *1174-901-12*_15901 | - | - | 0.59 |
| Monteiro hornbill | *Friedmanniella*_10715 | - | -0.90 | - |
| Monteiro hornbill | *Microbacterium*_9746 | - | 0.75 | - |
| Monteiro hornbill | *Sphingomonas*_17320 | - | 0.73 | - |
| Monteiro hornbill | *Blastococcus*_8751 | - | -0.71 | - |
| Monteiro hornbill | *Bradyrhizobium*_20106 | - | 0.71 | - |
| Red-billed hornbill | *Lactobacillus*_7496 | - | 0.90 | 0.90 |
| Red-billed hornbill | *Microbacterium*_9745 | - | -0.85 | -0.85 |
| Red-billed hornbill | *Hymenobacter*_4073 | - | 0.82 | 0.82 |
| Red-billed hornbill | *Staphylococcus*_7581 | - | -0.82 | -0.82 |
| Red-billed hornbill | *Staphylococcus*_7651 | - | 0.81 | 0.81 |
| Red-billed hornbill | *Sphingomonas*_17316 | - | -0.81 | -0.81 |
| Red-billed hornbill | *Solibacillus*_6826 | - | 0.80 | 0.80 |

**Figure S8.** Q² values for each sPLS model after validation. Leave-one-out cross-validation was used for models with fewer than 20 samples, while 5-fold cross-validation with 50 repeats was applied for models with 20 or more samples. For each model, sample sizes and the number of ASVs used as predictors are indicated above the plots (only ASVs present in at least 4 samples and representing more than 0.008% of total ASV counts). The y-axis represents Q² values, and the x-axis indicates components 1 and 2. The black line represents the established minimum Q² threshold of 0.095.

**References**

1.	Martín-Vivaldi M, Ruiz-Rodríguez M, Soler JJ, Peralta-Sanchez JM, Mendez M, Valdivia E, Martin-Platero AM, Martínez-Bueno M. Seasonal, sexual and developmental differences in hoopoe *Upupa epops* preen gland morphology and secretions: evidence for a role of bacteria. Journal of Avian Biology. 2009; 40:191-205.

2.	Burger BV, Reiter B, Borzyk O, du Plessis MA. Avian exocrine secretions. I. Chemical characterization of the volatile fraction of the uropygial secretion of the green woodhoopoe, *Phoeniculus purpureus*. Journal of Chemical Ecology. 2004; 30:1603-1611.

3.	Law-Brown J, Meyers PR. *Enterococcus phoeniculicola* sp. nov., a novel member of the enterococci isolated from the uropygial gland of the Red-billed Woodhoopoe, *Phoeniculus purpureus*. International Journal of Systematic and Evolutionary Microbiology. 2003; 53:683-685.

4.	Cameron ES, Schmidt PJ, Tremblay BJ-M, Emelko MB, Müller KM. Enhancing diversity analysis by repeatedly rarefying next generation sequencing data describing microbial communities. Scientific Reports. 2021; 11:22302.

5.	Lahti L, Shetty S. microbiome R package. 2017.

6.	Kembel SW, Cowan PD, Helmus MR, Cornwell WK, Morlon H, Ackerly DD, Blomberg SP, Webb CO. Picante: R tools for integrating phylogenies and ecology. Bioinformatics. 2010; 26:1463-1464.

7.	Dell-Inc. STATISTICA (data analysis software system), version 13. software.dell.com; 2015.

8.	Jetz W, Thomas GH, Joy JB, Hartmann K, Mooers AO. The global diversity of birds in space and time. Nature. 2012; 491:444-448.

9.	Kemp A, Delport W. Comments on the status of subspecies in the red-billed hornbill (Tockus erythrorhynchus) complex (Aves: Bucerotidae), with the description of a new taxon endemic to Tanzania. Annals of the Transvaal Museum. 2002; 39:1-8.

10.	Gonzalez JC, Sheldon BC, Collar NJ, Tobias JA. A comprehensive molecular phylogeny for the hornbills (Aves: Bucerotidae). Molecular Phylogenetics and Evolution. 2013; 67:468-483.

11.	Maddison WP, Maddison DR. Mesquite: a modular system for evolutionary analysis. Version 3.04 edn; 2015.

12.	Mazel F, Davis KM, Loudon A, Kwong WK, Groussin M, Parfrey LW. Is host filtering the main driver of phylosymbiosis across the tree of life? Msystems. 2018; 3:10.1128/msystems. 00097-00018.
